# Development of Web-Based Education Modules to Improve Carer Engagement in Cancer Care: Design and User Experience Evaluation of the e-Triadic Oncology (eTRIO) Modules for Clinicians, Patients, and Carers

**DOI:** 10.2196/50118

**Published:** 2024-04-17

**Authors:** Rebekah Laidsaar-Powell, Sarah Giunta, Phyllis Butow, Rachael Keast, Bogda Koczwara, Judy Kay, Michael Jefford, Sandra Turner, Christobel Saunders, Penelope Schofield, Frances Boyle, Patsy Yates, Kate White, Annie Miller, Zoe Butt, Melanie Bonnaudet, Ilona Juraskova

**Affiliations:** 1 Centre for Medical Psychology & Evidence-based Decision-making School of Psychology The University of Sydney Sydney Australia; 2 Psycho-Oncology Co-operative Research Group The University of Sydney Sydney Australia; 3 Flinders Medical Centre Adelaide Australia; 4 College of Medicine and Public Health Flinders University Adelaide Australia; 5 School of Computer Science The University of Sydney Sydney Australia; 6 Health Services Research and Implementation Science Peter MacCallum Cancer Centre Melbourne Australia; 7 Department of Radiation Oncology Westmead Hospital Westmead Australia; 8 Faculty of Medicine and Health The University of Sydney Sydney Australia; 9 Department of Surgery Royal Melbourne Hospital University of Melbourne Melbourne Australia; 10 Department of Psychology and Iverson Health Innovation Research Institute Swinburne University Melbourne Australia; 11 Sir Peter MacCallum Department of Oncology The University of Melbourne Melbourne Australia; 12 Patricia Ritchie Centre for Cancer Care & Research Mater Hospital Sydney Australia; 13 Faculty of Health Queensland University of Technology Brisbane Australia; 14 Susan Wakil School of Nursing, The Daffodil Centre The University of Sydney, a joint venture with Cancer Council New South Wales Sydney Australia; 15 Cancer Council New South Wales Sydney Australia; 16 School of Electrical Engineering and Computer Science Kungliga Tekniska högskolan Royal Institute of Technology Stockholm Sweden

**Keywords:** family carers, patient education, health professional education, web-based intervention, mobile phone

## Abstract

**Background:**

Carers often assume key roles in cancer care. However, many carers report feeling disempowered and ill‐equipped to support patients. Our group published evidence-based guidelines (the Triadic Oncology [TRIO] Guidelines) to improve oncology clinician engagement with carers and the management of challenging situations involving carers.

**Objective:**

To facilitate implementation of the TRIO Guidelines in clinical practice, we aimed to develop, iteratively refine, and conduct user testing of a suite of evidence-based and interactive web-based education modules for oncology clinicians (e-Triadic Oncology [eTRIO]), patients with cancer, and carers (eTRIO for Patients and Carers [eTRIO‐pc]). These were designed to improve carer involvement, communication, and shared decision-making in the cancer management setting.

**Methods:**

The eTRIO education modules were based on extensive research, including systematic reviews, qualitative interviews, and consultation analyses. Guided by the person-based approach, module content and design were reviewed by an expert advisory group comprising academic and clinical experts (n=13) and consumers (n=5); content and design were continuously and iteratively refined. User experience testing (including “think-aloud” interviews and administration of the System Usability Scale [SUS]) of the modules was completed by additional clinicians (n=5), patients (n=3), and carers (n=3).

**Results:**

The final clinician module comprises 14 sections, requires approximately 1.5 to 2 hours to complete, and covers topics such as carer-inclusive communication and practices; supporting carer needs; and managing carer dominance, anger, and conflicting patient-carer wishes. The usability of the module was rated by 5 clinicians, with a mean SUS score of 75 (SD 5.3), which is interpreted as good. Clinicians often desired information in a concise format, divided into small “snackable” sections that could be easily recommenced if they were interrupted. The carer module features 11 sections; requires approximately 1.5 hours to complete; and includes topics such as the importance of carers, carer roles during consultations, and advocating for the patient. The patient module is an adaptation of the relevant carer module sections, comprising 7 sections and requiring 1 hour to complete. The average SUS score as rated by 6 patients and carers was 78 (SD 16.2), which is interpreted as good. Interactive activities, clinical vignette videos, and reflective learning exercises are incorporated into all modules. Patient and carer consumer advisers advocated for empathetic content and tone throughout their modules, with an easy-to-read and navigable module interface.

**Conclusions:**

The eTRIO suite of modules were rigorously developed using a person-based design methodology to meet the unique information needs and learning requirements of clinicians, patients, and carers, with the goal of improving effective and supportive carer involvement in cancer consultations and cancer care.

## Introduction

### Background

Carers (including but not limited to spouses, partners, adult children, siblings, parents, or friends [1]) of adults with cancer assume many responsibilities in supporting and caring for their loved one [2]. Carers can experience many challenges in this demanding role and often report high distress [3,4], poor physical health, low quality of life, and unmet needs [5,6]. As carer burden increases, carers may neglect their own needs, which can also impact their ability to support and care for their loved one [7,8].

While issues faced by carers are well recognized by health care professionals [9], many clinicians report that they do not know how to appropriately engage with carers or address their unique challenges [9,10]. Oncologists have reported a lack of education about communicating with carers [10], and suboptimal carer-clinician communication is common [11]. Some carers report being overlooked in medical consultations and feeling disempowered and unprepared in their caregiving role [12]. Clinician inclusion and support of carers have been reported as highly valued by both carers and patients [12].

Improving carer engagement and support needs to be addressed from multiple perspectives. Not only are clinicians uncertain about how to include carers in consultations [9] but also many carers often lack confidence and skills in caregiving [12,13], and some patients are unsure about what role their carer should assume in medical consultations and decision-making [14]. Therefore, interventions targeting *all* members of the clinician-patient-carer trio are needed.

Web-based delivery of education offers efficacy, efficiency, ability to undertake training in discrete periods, lower cost, flexibility, and greater reach than traditional face-to-face formats [15]. A systematic review of web-based health education by George et al [16] found web-based training for health professionals to be as effective as or better than face-to-face formats on outcomes such as knowledge, skills, and attitudes. Web-based communication skills interventions have been found to be effective in improving self-rated clinician confidence, communication skills, and knowledge among cancer clinicians [15]. For example, a web-based module developed by our group to educate nurses about managing conflict involving patients and carers (the Triadic Oncology [TRIO]–Conflict module) was found to improve cancer nurses’ attitudes and confidence in interacting with carers [17].

Patients and carers can also benefit from web-based resources and educational tools [18]. A systematic review of digital psychosocial interventions for patients with cancer and carers found web-based interventions to be both feasible and acceptable [19]. Digital interventions for carers have been shown to improve carer outcomes, knowledge, and skills, with the additional benefit of being accessible from home, thus minimizing the demands on carers’ time [20]. For example, a web-based psychosocial intervention for patients with cancer, Stress-Aktiv-Mindern (STREAM), has demonstrated beneficial patient outcomes including reduced stress and improved quality of life [21]. Similarly, the psychoeducational platform, Comprehensive Health Enhancement Support System (CHESS), has demonstrated favorable outcomes among carers such as significant reduction in negative mood and carer burden [22]. These beneficial effects were comparable to those of traditional psychoeducation interventions [23,24]. While STREAM and CHESS demonstrate the efficacy of web-based patient and carer support, their focus is on *psychosocial* support. To date, there have been no web-based education modules dedicated to empowering and upskilling patients and carers in *carer-relevant* *communication and engagement* with cancer clinicians and in carer participation in cancer treatment decision-making. Therefore, we aimed to develop and evaluate a web-based learning tool to address these needs.

Interventions to support cancer carers are often difficult to implement in clinical practice and face barriers to implementation including problems with design, feasibility, acceptability, and cost [25]. One way to improve the acceptability and sustainability of an intervention is to use a co-design approach with the target population as stakeholders, to ensure that the program targets user needs and preferences. The person-based approach [26] ensures that intervention development is grounded in the perspectives and psychosocial context of end users via iterative, qualitative research with relevant stakeholders. This approach has been effectively used in the development of web-based health care interventions [27,28].

### Objectives

This paper describes the development, iterative refinement, and user testing of evidence-based and interactive web-based interventions designed to improve engagement and communication with carers in cancer care. We have published the study protocol for a randomized controlled trial to test the efficacy of the e-Triadic Oncology (eTRIO) modules elsewhere [29]. However, necessary amendments to the planned randomized controlled trial due to the COVID-19 pandemic were made after publication of the protocol. The evaluation approach was revised to hybrid effectiveness and implementation studies using a pre-post, single-arm intervention design.

In this paper, we have reported about the development of web-based education modules for all 3 relevant stakeholder groups, including oncology health professionals and patients with cancer and carers (eTRIO for patients and carers [eTRIO-pc]).

## Methods

### Overview

**Figure 1:**
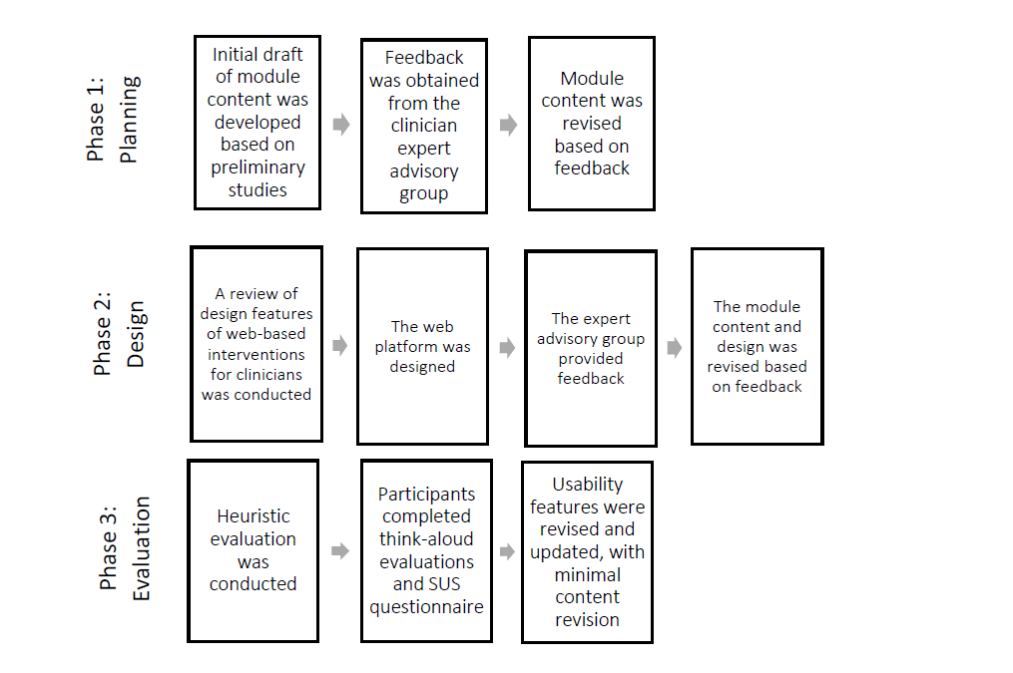
e-Triadic Oncology (eTRIO; clinician) module development process. SUS: System Usability Scale.

**Figure 2:**
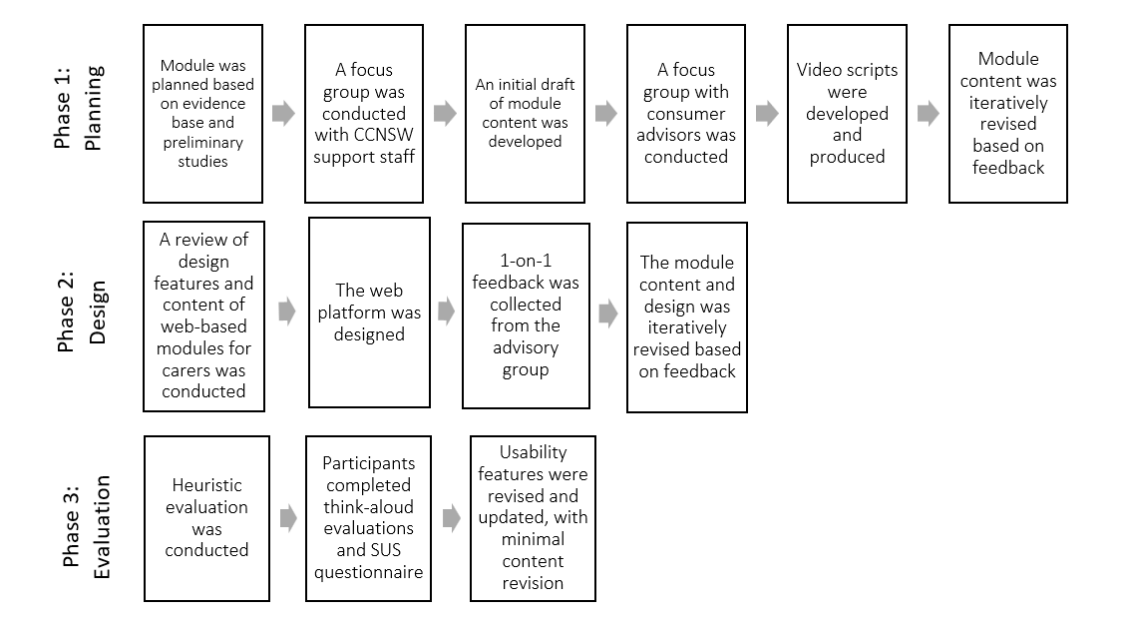
e-Triadic Oncology for patients and carers (eTRIO-pc) module development process. CCNSW: Cancer Council New South Wales; SUS: System Usability Scale.

The person-based co-design approach by Yardley et al [26] underpinned the module design. Development and user experience testing of the clinician (eTRIO) and patient-carer (eTRIO-pc) modules was undertaken in multiple cyclical phases of data collection, analysis, and integration, in a process of iterative refinement [30]. Consistent with the approach by Yardley et al [26], this involved (1) *planning*: development of module content based on evidence, qualitative interviews with stakeholders, and input from our expert advisory group; (2) *design*: iterative review and refinement based on advisory group feedback; and (3) *development and evaluation of acceptability and feasibility*: formal heuristic evaluation, System Usability Scale (SUS) questionnaire, and think-aloud review of the eTRIO modules by stakeholders (Figures 1 and 2). The final phase of implementation and trialing is currently being conducted in a separate pre-post evaluation study, which will be reported elsewhere.

### Phase 1: Development of eTRIO and eTRIO-pc Module Content

#### Development of the eTRIO Clinician Module

The content of the modules was informed by our extensive Triadic Oncology (TRIO) research program, which includes a systematic review of carer involvement in consultations [31]; qualitative interviews with oncology clinicians, patients, and carers [9,14,32]; quantitative and qualitative analyses of audiotaped oncology consultations [11]; a conceptual framework of carer involvement in medical decisions [33]; and carer communication guidelines for clinicians (TRIO Guidelines) developed via a Delphi consensus process [34,35]. Key clinician training needs, strategies, and behaviors relevant to the module were ascertained through this extensive research program.

On the basis of this prior research, we developed an initial draft of the eTRIO content. The draft module comprised 14 study sections (1 introductory section and 13 strategy areas covered in the TRIO Guidelines [34,35]). A clinician expert advisory group was formed to provide feedback about the module content, comprising medical oncologists (3/13, 23%), oncology nurses (2/13, 15%), psychologists (2/13, 15%), a radiation oncologist (1/13, 8%), an oncology surgeon (1/13, 8%), and the research team comprising psycho-oncologists (4/13, 31%). Each member of the clinician expert advisory group reviewed a text-based draft of the module content and provided written feedback about each module section, including interactive activities, reflective exercises, and wording of strategies. Multiple teleconferences were conducted, where group members provided feedback about the content and structure of each section. Major changes were discussed with the group until consensus was reached. Feedback from the advisory group was collated, and the module content was iteratively refined.

#### Development of the eTRIO-pc Patient-Carer Module

The eTRIO-pc module content was drafted based on a review of current web-based guidance for carers about involvement in medical consultations [18], qualitative studies of patients and carers [9,14,32], and analyses of audiotaped consultations [11]. A meeting with the staff at a leading nongovernment cancer support and advocacy organization (n=5) was also conducted to inform the content of the eTRIO-pc initial draft. The staff members were asked to describe the key content that should be included in the eTRIO-pc module, based on their experience in supporting patients and carers via a telephone information and support service.

Consumer advisers (3/5, 60% cancer carers and 2/5, 40% patients with cancer) also provided iterative feedback about the module content during a half-day workshop and via email. Consumer advisers were asked to comment about whether the module content was understandable, the relevance of the module content and feasibility of the suggested strategies, the language, and tone of the module. All feedback from the Cancer Council New South Wales support staff and consumer advisers was collated and discussed with the project team until consensus was reached through revisions.

After the development and iterative revision of the module content was complete, video vignettes modeling key carer communication skills were developed to supplement the written content. Video vignettes have been demonstrated as an effective educational tool for patients and carers and can improve accessibility for those with low literacy [36,37]. We engaged a professional medical education and communication production company to develop a script covering key learning areas for carers, as determined by the consumer advisory groups. The script was iteratively reviewed by the research team, consumer advisers, and a physician to ensure that the videos aligned with the TRIO communication guidelines [34,35] and were clinically relevant and feasible.

### Phase 2: Iterative Design, Review, and Refinement of eTRIO and eTRIO-pc Web-Based Modules

#### Design and Refinement of eTRIO Clinician Module

As shown in Figure 1, phase 2 involved consumer input and refinement of the modules. To translate the text-based content into an interactive web-based educational module, we studied the best practice principles for the delivery of e-learning to health professionals [16,38-40]. This included a review by de Leeuw et al [38] about e-learning features targeted at postgraduate medical students and health professionals completing ongoing professional development, which identified 6 domains of important elements for e-learning quality (preparation, design, communication, content, assessment, and maintenance). Informed by a previous review [38], we developed a base design and catalog of potential design features.

A prototype web platform was developed by a professional web development company. In 2 sessions conducted via Zoom (Zoom Video Communications), the clinician advisory group completed a walk-through of the module and provided comprehensive feedback. Their verbal and written feedback was collated and integrated into a revised web-based module.

#### Design and Refinement of the eTRIO-pc Patient-Carer Module

Similarly, as displayed in Figure 2, phase 2 involved the conversion of the text-based module content for patients and carers into an interactive web-based platform. We conducted a review of the content and design features of other available evidence-based web-based platforms for carers [18], drew on the evidence base surrounding education for carers [41-43], and received input from the consumer advisory group. To inform the website design, we reviewed the publicly available web-based resources for carers.

The final design features of eTRIO and eTRIO-pc were implemented by a professional web development company and included interactive activities, video vignettes, and text-based content. The clinician and consumer advisory groups were given access to the draft module, and its content and format were revised based on their extensive feedback. An expert in human-centered IT design was involved in all stages of development of the clinician and patient-carer modules.

### Phase 3: Heuristic Evaluation and “Think Aloud” User Experience Evaluation of eTRIO and eTRIO-pc Web-Based Modules

As shown in Figures 1 and 2, phase 3 involved usability evaluations of the developed web-based module. We conducted a heuristic evaluation to discover technical and usability issues [44]. The modules were examined by the researchers to identify problems that did not comply with the usability principles recognized by Nielsen [45], which include consistency and standards, error prevention, and aesthetic and minimalist design. The severity and prevalence of the issues were ranked from 1 to 5, with a high rank indicating that the problem was a priority to fix, and the web platform was updated accordingly.

Usability and user experience testing for the penultimate versions of eTRIO and eTRIO-pc were conducted using think-aloud methodology with 11 participants, including clinicians, patients, and carers, all of whom were naïve to the TRIO Guidelines and modules. Think aloud is an effective evaluation method in which participants are provided with an interface and asked to verbalize their thoughts as they work through it [46,47]. Potential participants were identified through the research team’s professional networks and via social media advertisements.

The consenting participants completed a demographic questionnaire and a 4-item self-report measure of health literacy [48]. Participants were provided access to the relevant eTRIO module and asked to speak aloud their thoughts and impressions as they were completing the module (think-aloud). These sessions were conducted face to face or via videoconferencing. After working through the module, participants completed the SUS [49]. Think-aloud evaluations were audio recorded and transcribed verbatim. Transcripts were qualitatively analyzed using thematic analysis [50], which involved familiarization with the transcripts, coding of salient initial ideas as codes, identification of patterns in the codes to generate themes and subthemes, and iterative review of the themes and subthemes to ensure a coherent and comprehensive thematic structure. This process was conducted collaboratively and through iterative discussion by RLP, PB, ZB, MB, and IJ. Themes were related to the following: usability and technical issues, positive aspects of design and function, attitudes toward the content of the program, and perspectives about the impact or implementation of the program. All transcripts were analyzed based on the established thematic framework and were grounded in illustrative quotations. Subsequently, the modules were iteratively refined based on this feedback.

### Ethical Considerations

Ethics approval was obtained from the University of Sydney Human Research Ethics Committee (protocol 2015/468). Participants provided informed consent and were given the opportunity to opt out at any point in time. Participant data were deidentified. Participants were provided a gift card worth Aus $20 (US $13.22) as compensation for their time.

## Results

This section describes the clinician, patient, and carer feedback; iterative revisions made; and lessons learned in the design and development of the eTRIO and eTRIO-pc modules.

### Phase 1: Development of eTRIO and eTRIO-pc Module Content

#### eTRIO Clinician Module

The clinician advisory group members (n=13) emphasized the importance of the module being concise. They suggested more content for the introductory section such as including a broad and inclusive definition for “carers,” content about culturally diverse carers, and more information about the legal and ethical aspects of involving carers. Clinicians also suggested the inclusion of self-reflections about one’s own attitudes and potential biases toward carers. Additional suggestions included addressing the diversity of settings in which family or carer interactions can occur (eg, outside traditional outpatient consultations such as at the patient’s bedside or via the telephone). Several clinicians stressed the importance of including clear learning outcomes and summaries for each of the 14 sections.

#### eTRIO-pc Patient-Carer Module

Cancer support staff (n=5) suggested a clear definition of the role of carers, tailoring based on the cultural backgrounds of patients and carers, and consideration of power imbalances that may exist in patient-carer relationships. They emphasized checking in on patient and carer emotions such as grief and distress, suggested that modules could include opportunities for self-reflection, and highlighted the need to include information about available support for carers.

The overall impression of the consumer advisory group (n=5) was that the language and tone of the draft module was very formal and academic; they wanted the tone to be more “personal,” “empathetic,” and “softer” and the language to be less prescriptive. They suggested additional strategies for patients with newly diagnosed cancer and carers, such as making notes during medical consultations, and suggested including quotes and stories from actual carers to illustrate examples.

### Phase 2: Iterative Design, Review, and Refinement of eTRIO and eTRIO-pc Web-Based Modules

#### Overview

Table 1 describes the results from phase 2 using the e-learning design features by de Leeuw et al [38] applied to the eTRIO and eTRIO-pc modules.

**Table 1:** e-Learning design features identified by de Leeuw et al, as applied to the e-Triadic Oncology (eTRIO) and e-Triadic Oncology for patients and carers (eTRIO-pc) modules.

Elements of e-learning	Description	Use in eTRIO and eTRIO-pc
Preparation	Identifying the needs of the target audience	Th research team conducted an extensive program of previous studies on the needs of carers Stakeholder input, feedback, and evaluation
Design	Including elements of accessibility, reliability, user-friendly navigation, and visual appeal	Web-based program, simple layout, and designed for easy use Font, color, size, and layout are optimized for accessibility User progress is saved when users log out Website is designed and tested on various software and hardware
Communication	Communication with users and program facilitators	Landing page introduces users to the learning objectives and goals (ie, communication skills and strategies, understanding carer roles, and benefits of carer involvement in cancer care) Clear information about program use and navigation is included
Content	Including words, images, videos, interactive activities, summaries, and so on	All modules include multimedia content such as several clinical vignette videos, audios, text, images, and interactive features. Interactive activities were designed, including the following: *eTRIO (clinician)*: sorting and drag-and-drop activities, true-or-false exercises, open-text written responses, click-to-expand sections, and identifying behaviors in a vignette video *eTRIO-pc (patient-carer)*: resources that can be individually tailored (eg, assembling a caregiving team, building a question prompt list, and checklist for patients and carers to discuss carer role), click-to-expand sections, and open-text written responses Downloadable summaries are provided to allow access after completing or outside the module
Assessment	Assessing learning and acquiring feedback	Each section of each module contains clear learning objectives, displayed on the first page of each section. For example, section 9 of eTRIO (clinician), related to the use of interpreters, states the following: “In this section you will explore reasons why patients/carers might resist professional language interpreters, and understand strategies to overcome these issues. You will learn practical strategies to engage and use formal interpretation services.” All modules include assessment activities to facilitate learning and reflection: *eTRIO (clinician)*: self-reflection and assessment of own attitudes and practices, true-or-false assessment of content with correct answers and explanations, multiple choice questions asking users to reflect about how they would navigate a clinical scenario, and open-ended responses *eTRIO-pc (patient-carer)*: self-assessment of emotions, opportunities to reflect about own preferences and attitudes and to plan future actions or behaviors, and open-text reflections about video vignettes modeling key skills
Maintenance	Providing long-term access and updating information and links	Website is regularly maintained and updated All users will have access to the program after completion of the training

#### eTRIO Clinician Module

During the transformation of content to a web-based module, features of e-learning [38] were applied as described in Table 1. The design features of other web-based clinician training modules were examined, revealing display, navigation, and interactive activity styles (eg, minimal use of text, prominent navigation buttons, and clickable and expandable content). Our team worked closely with graphic and web designers to develop a consistent color scheme and intuitive navigation system and aimed to minimize visual noise on each page. The refined content and design features were transformed into a web-based web platform.

All members of the clinician advisory group (13/13, 100%) commented that there was excessive content and that there would not be clinician appetite for web-based training that extended beyond 2 hours in total. The content was subsequently condensed, with the core content displayed with the option of more extensive content, which could be expanded for clinicians interested in deeper learning regarding an issue.

The final eTRIO clinician module comprises 14 sections (submodules), of which clinicians must complete a minimum of 8. The sections range between 3 and 15 minutes in duration. The following 4 sections were deemed to be mandatory by the clinician advisory group, based on their critical relevance to all clinicians: section 1—introduction, section 4—building rapport with carers, section 7—supporting carers’ emotional and informational needs, and section 10—managing conflicting patient-carer treatment preferences. Clinicians could select additional 4 sections based on their interest and preference. The eTRIO module requires approximately 1.5 to 2 hours to complete, as determined by multiple stakeholders working through the content and documenting the amount of time each section required to complete.

#### eTRIO-pc Patient-Carer Module

Consistent with the principles of computer-based teaching for adult learners by Lau [51], the web-based eTRIO-pc module was created by transforming the written content into interactive, engaging learning activities. Our review of carer resources demonstrated several useful stylistic, formatting, and usability features, for example, the use of bullet points to convey written information, 1-page displays eliminating the need to scroll, and use of simple navigation buttons. These features and principles of web-based education were collated and discussed with the team’s academic IT expert and web developers to select and finalize the most appropriate features to be included. The resultant module prototype included video vignettes that could easily be played and paused, interactive activities such as “drag-and-drop” and “click to reveal” exercises, and type-your-response activities (Multimedia Appendix 1). We maintained consistency in design and formatting across the clinician, patient, and carer modules.

We sent the prototype to the members of the consumer advisory group (n=5), and they provided written feedback via email and offered additional personal quotes that could be included in the module to personalize the content. They re-emphasized the need for content that was empathetic and offered practical advice. The final eTRIO-pc modules contain 7 sections for patients and 11 sections for carers and requires approximately 1 to 1.5 hours to complete.

### Phase 3: “Think Aloud” Usability Evaluation of eTRIO and eTRIO-pc Web-Based Modules

#### Heuristic Evaluation

Using the heuristic evaluation method [44], we identified 37 usability issues across the draft eTRIO and eTRIO-pc modules, and each was rated for severity. The main areas of the identified problems were as follows: (1) inconsistency of icons and redundancy in buttons (5/37, 14% of the issues; eg, inconsistent use of star and book icons to indicate the bookmark function), (2) buttons and interactions were not working (16/37, 43% of the issues; eg, nothing happens when the print button is clicked), (3) layout problems (6/37, 16% of the issues; eg, text is not aligned with the textbox), and (4) presentation of content (10/37, 27% of the issues; eg, color selection in the bar-slider activity may be confusing) [52]. Following this evaluation, problems with high severity and prevalence were prioritized, and all issues that could be corrected were fixed before conducting the think-aloud user evaluations.

#### Think-Aloud User Experience Evaluations

Overall, 11 individuals (n=5, 45% health professionals; n=3, 27% patients; and n=3, 27% carers) participated in the think-aloud evaluations in individual sessions lasting between 40 and 60 minutes. Participant characteristics are displayed in Table 2.

**Table 2:** Characteristics of participants of the think-aloud evaluations.

Participant category and characteristics				Values
**Health professionals (n=5)**				
	Age (y), mean (SD; range)		47 (10.3; 35-58)	
	**Sex, n (%)**			
		Female	4 (80)	
		Male	1 (20)	
	**Profession, n (%)**			
		Physician	2 (40)	
		Nurse	3 (60)	
	**Clinical expertise, n (%)**			
		Oncology	2 (40)	
		Palliative care	2 (40)	
		Geriatrics	1 (20)	
	Experience (years), mean (SD; range)		22 (9.8; 12-37)	
**Patients (n=3)**				
	Age (y), mean (SD; range)		65 (13.7; 50-77)	
	Sex (female), n (%)		3 (100)	
	**Diagnosis, n (%)**			
		Kidney cancer	1 (33)	
		Colorectal cancer	1 (33)	
		Non-Hodgkins lymphoma	1 (33)	
	**Cancer stage, n (%)**			
		Local	2 (67)	
		Advanced	1 (33)	
	**Health literacy, n (%)**			
		Low	1 (33)	
		Medium	1 (33)	
		High	1 (33)	
**Carers (n=3)**				
	Age (y), mean (SD; range)		65 (8.7; 58-75)	
	**Sex, n (%)**			
		Female	2 (67)	
		Male	1 (33)	
	**Relationship with care recipient, n (%)**			
		Spouse or partner	2 (67)	
		Mother	1 (33)	
	**Diagnosis of care recipient, n (%)**			
		Lung cancer	1 (33)	
		Multiple myeloma	1 (33)	
		Non-Hodgkins lymphoma	1 (33)	
	**Cancer stage of care recipient, n (%)**			
		Local	1 (33)	
		Advanced	2 (67)	
	**Health literacy, n (%)**			
		Medium	1 (33)	
		High	2 (67)	

#### eTRIO Clinician Module

The usability of the module was rated by 5 clinicians, with a mean SUS score of 75 (range 68-80), which is interpreted as good [49]. All clinicians gave high ratings to their ability to use the module independently without technical assistance. Clinicians identified technical and navigation issues, which were subsequently rectified (such as the side scroll bar not appearing, text appearing outside the text bubble, and a sliding bar not working responsively). For some, the use of specific web browsers corrected these issues. Clinicians described the overall navigation through the module as “straightforward.” Formatting issues with font size and background color were highlighted. Clinicians commented that the ability to easily navigate back to certain sections to “refer back to later” was valued.

Content analysis of think-aloud evaluations revealed 7 categories related to clinicians’ attitudes toward the design and formatting of eTRIO. Clinicians appreciated that the modules could be completed in small “snackable” periods in any order, that they could keep track of what sections were completed (*trackable*), and that they were able to refer back to any module at any time*.* Clinicians enjoyed the “clickable” activities where they interacted with the content. Despite attempts to make the sections as short as possible (average 5-10 min/section), a few clinicians still perceived them as “too long,” with some stating that the videos were “slow” at times. They highlighted a preference for material that is brief, uses simple language, is easy to digest, and “skimmable.” A few clinicians reported “glossing over” or “tuning out” when sections were perceived as very long. They suggested simplifying the language and formatting the text to highlight important information (eg, use of bullet points and bold and italic style). Revisions were made to the text to further improve conciseness, including rephrasing the core content, moving some content to the expandable ‘additional information’ section, and greater use of bullet points and bold text. Where possible, videos were edited to remove nonessential scenes. Most participants appreciated that the content and activities were relevant and “relatable” to them as clinicians, that claims were “supported” by evidence, and that the activities and media were “diverse” and varied to facilitate engagement and interest. Illustrative quotes are provided in Table 3.

**Table 3:** Illustrative quotes from think-aloud evaluations by clinicians.

Usability and content feature	Description	Illustrative quotes
Snackable	Ability to complete the module in small segments	“So, you’re saying you don’t have to do it all in one go...oh, I think that’s really important because you do get called away and the phone is ringing...because I know even with our mandatory online training in the past, you just [had to] forfeit [all progress] if you couldn’t finish.” [Nurse 2]
Trackable	Ability to know what has been completed and refer to the content later	“It’s nice to have things you can refer back to because this might trigger things that make you think oh yeah, I did read about that.” [Physician 2]
Clickable	Importance of interactive content	“I like this section - it’s really good. I like that activity. I’ve never done one of those before - that’s really good. [Interactive activity clicking points of rapport building throughout a video vignette]. You definitely engage a thousand percent more with the activities.” [Physician 2] “I think [the activities] are quite good because at least you are giving people a little bit more of themselves...I think it’s good to have that interaction rather than just reading...that gets a bit boring. And then, that you ask people to actually write something is good.” [Nurse 2]
Skimmable	Importance of simple, concise language	“After reading articles all day I don’t want to read something that has too much jargon in it...Go back and simplify the language...when I read something apart from patients notes, I skim it. So, it’s got to be something that I can get the message with a glance.” [Physician 1] “Uhm why I am I finding it difficult to understand? I think it could be worded more simply.” [Physician 2] “Yeah. I hate the time pressure...It’s so built into our working day, it’s like get on, get it done, that you gloss over so much. I actually didn’t realize before doing this how much I gloss over...I probably would watch [the video] to the end but there’s a part of me thinking yeah it’s going on a little bit.” [Physician 2]
Relatable	Relevance of content to the user	“Yeah, I like that there is the suggestions of things to say. That makes it really relatable - I think those are good.” [Physician 2] “I like scenarios...Just sort of triggers you to think a little bit more rather than just reading through something. I think the scenario allows me to put it into practice or put it into place a little bit more.” [Nurse 3]
Supported	Evidence-based content	“I like the use of the quotes. It gives a bit of a supportive evidence to it, as nurse I like that...It has got some stats [statistics] there...When you hover over it...it gives the reference.” [Nurse 3]
Diverse	Importance of variety in media and activities	“Oh a video, that’s interesting, it’s sort of mixing it up, it’s nice to have the different things.” [Physician 2]

#### eTRIO-pc Patient and Carer Module

The average SUS score as rated by 6 patients and carers was 78 (SD 16.2; range 55-97.5), which is interpreted as good [49]*.* Patients and carers were generally happy with the content and usability of the eTRIO-pc module. They commented that the content was relatable and were pleased by the emphasis placed on carers. Overall, they found the web platform easy to navigate and enjoyed the interactive activities; however, 1 (17%) of the 6 patients found the interface to be “overwhelming.” A major critique of the formatting and layout was that the pages were “too busy” and contained excessive information. Illustrative quotes are provided in Table 4.

The final eTRIO and eTRIO-pc modules were updated based on this feedback. All technical and navigation issues were addressed by the web developers.

For both modules, the text was condensed and reformatted with the use of bold and italic style to highlight the important points and allow for easier reading and a more streamlined user interface.

**Table 4:** Illustrative quotes from think-aloud evaluations by patients and carers.

Usability and content feature	Description	Illustrative quotes
Snackable	Ability to complete the module in small segments	“Looking at this dashboard I like it that it tells you how long each part is going to take just so you know in advance. You’re busy and maybe you just have time to do half of it and then you can sort of plan how you’re going to tackle it.” [Carer 1]
Clickable	Importance of interactive content	“Some of the activities like the questions, I really liked. The ones where you wrote down what you thought the carer might do for you if you then use it as a communication tool, really good as well.” [Patient 1]
Usable	Ease of navigation	“I think [navigation] is pretty easy and straight forward. I think anybody who’s used to doing online training, modules and so on will probably find it really easy.” [Carer 1]
Relatable	Relevance of content to the user	“I think this is a very useful slide. When we went in to our first meeting we were just there, me and my son did this.” [Carer 1]
Visually simple	Cleanness of layout, formatting, and images	“I think you are making this page very busy with text and it’s a bit confronting.” [Carer 2] “It is pretty text-heavy and I guess that I am more of a visual learner so it might be nice to have some more pictures, icons, to make it a little bit more visually appealing.” [Patient 1]

#### Final Web Platform Design and Content Summary

The eTRIO modules reflect the reported informational needs of health professionals, patients with cancer, and carers. A full description of the module content has been published elsewhere [29]. The eTRIO modules have been rigorously designed to be easy to use, require minimal time commitment, and be flexible in terms of when and how the platform can be used. The modules are optimized for use on a computer but can also be used on a smartphone or tablet. Some notable features include the following: navigation buttons and a progress bar along the bottom of the page, expandable content for those who want deeper information about a specific topic, and downloadable summaries and lists. Notable interactive activities include the following: testing of knowledge through true-or-false exercises, identifying specific behaviors in a short video vignette, and building a question prompt list. Refer to Table 5 for descriptions and images of key features; full explanations of the interactive activities are provided in Multimedia Appendices 1 and 2.

**Table 5:** Key features of the e-Triadic Oncology (eTRIO) modules.

Feature	Description	Images
Interactive activities	Includes self-reflection, knowledge tests, and free-text responses	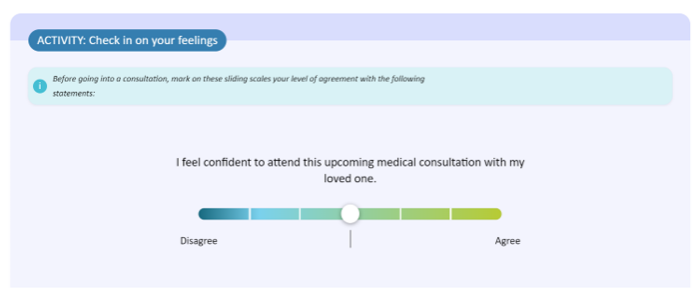
Learning outcomes	The eTRIO clinician module features signposted learning outcomes at the beginning of each section	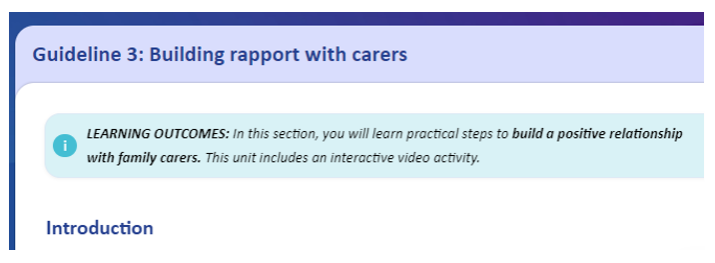
Downloadable content	Includes materials and personalized checklists for patients and carers and downloadable summaries for clinicians	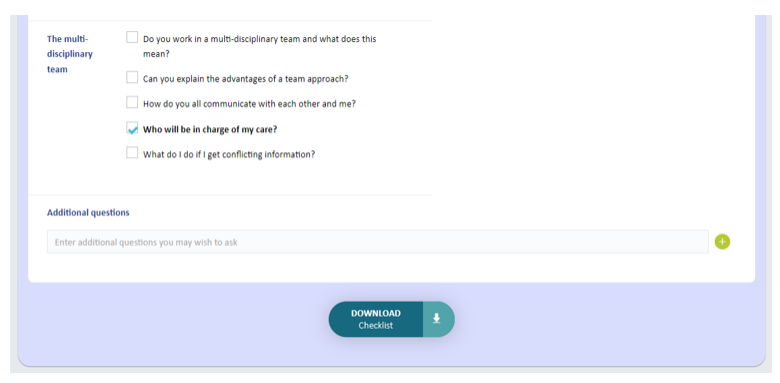 and 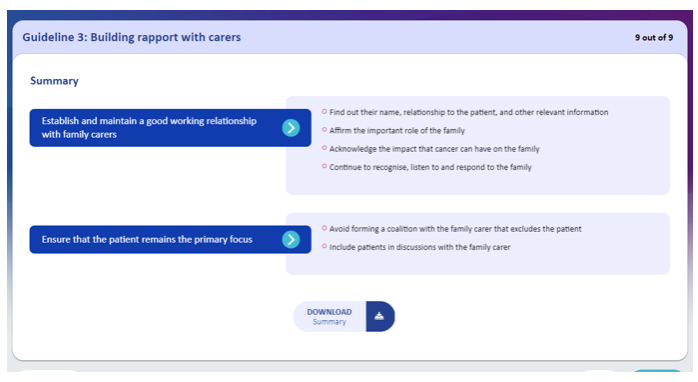
Video vignettes	Realistic scenarios modeling communication skills	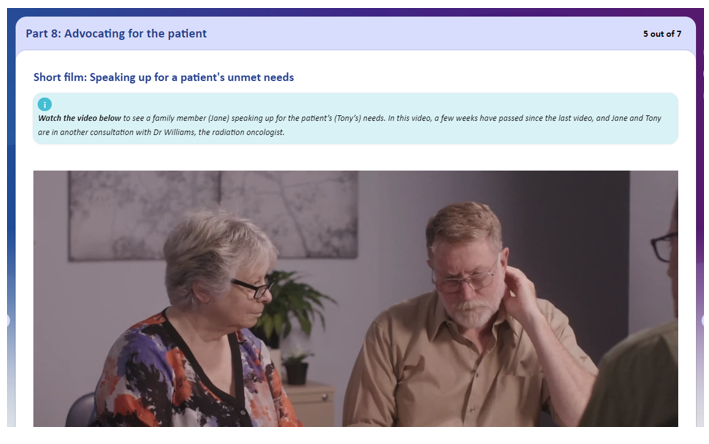
Intuitive navigation features	Navigation buttons are explained in the module’s introduction	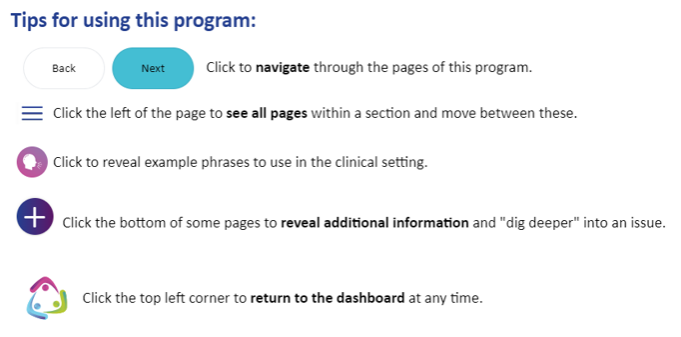

## Discussion

### Principal Findings

The web-based modules described in this paper represent a crucial step in the development and design of education for clinicians, patients, and carers that is evidence based, practical, and interactive and can be easily disseminated. Drawing on the evidence for best practice web-based learning design [38,51], we sought input from a variety of stakeholders to develop a unique learning experience strongly informed by the needs of the target populations. Rigor was ensured via 3 stages of development in which module content and design were continually revised and refined. Overall, participants were positive about the content and interface. The final prototype was appraised as highly acceptable, relevant, and feasible among the small sample of users; however, more studies are needed to confirm this and to ascertain the effectiveness of the intervention. We are currently conducting a pre-post evaluation of these modules to explore their potential effectiveness in improving communication within the patient-carer-clinician trio.

### Lessons Learned

Throughout the development and design of these modules, we observed the specific needs and preferences of end users. The person-based approach to developing eTRIO and eTRIO-pc was highly dynamic, and the modules underwent numerous iterations throughout all phases of the design process, which included the involvement of consumers and user-driven evaluations. While there are multiple approaches to developing health interventions, the benefits of the person-based approach include grounding the design in user contexts and lived experiences, integrating feedback based on the actual use of an intervention, and investigating user needs and perspectives beyond just the usability of the intervention [26]. The utility of the person-based approach has been extolled in recent studies [53-55] and is supported by the findings of this study. The eTRIO development process (Figures 1 and 2) provided the necessary building blocks to revise and refine the module for effective use in the real world. Consistent with other studies [56,57], we found that the collaborative co-design process led to positive evaluations of acceptability and usability and high levels of end-user satisfaction.

As highlighted in the person-based approach, the 3 user groups (clinicians, patient, and carers) demonstrated diverse learning preferences and needs. This was accommodated via tailoring the formatting or content to the strengths and contextual demands of different user groups and differentiating the content based on user needs. We found that clinicians had a strong desire for content that was written in simple, concise, and “sharp” language; could be “skim read”; and could be completed in brief, “snack-sized” sections. For example, clinicians in our advisory group often stressed that they lacked time and that training needed to be short, precise, and able to be stopped and restarted due to interruptions. On the other hand, the structure and time demands of training appeared to be less important to patients and carers. Instead, these groups emphasized the need for the module to be easy to use and navigate and for the content to be more conversational, empathetic, and in plain language (in contrast to the preferences of clinicians). Clinicians in our study valued the integration of academic literature and referencing, whereas some carers advocated for greater inclusion of carer experiences and quotes. The preferences of carers in our study are consistent with previous studies, which have similarly found that carers often prefer web-based education to have an empathetic and supportive tone, the web program to be easy to navigate, and the integration of other carers’ experiences into the content [58-60]. While several differences were identified between the clinician and carer user groups, there were also several similarities across all user groups in how the web-based modules should be structured and delivered. This is reflected in the evidence base, where health professionals, patients, and carers alike report that they prefer flexible, self-paced delivery of web-based programs that are interactive and include a variety of activities across media (visual, written, and auditory) [19,38]. These detailed insights are valuable in designing future training modules to facilitate their acceptability among users in each specific group.

The final interface used design principles to ensure engaging and interactive content. There is robust empirical evidence suggesting that interactivity in e-learning improves quality, efficacy, and learning outcomes [38,61]. For example, users of a web-based public health program had better learning outcomes when they used a gamified, interactive version featuring responsive design, learning challenges, visible progress, and rapid feedback compared to those using a minimally interactive, survey-based program [62]. Such interactivity was also demonstrated as important for users of the eTRIO modules. For example, in the initial design phases, when content was largely text based, the advisory committee members noted how dense the information appeared. While this was never intended to be the final format of the educational intervention, comments obtained from users in phase 1 highlighted the limitations of passive, didactic, text-heavy information. There is evidence suggesting that people do not learn effectively when information is given without any opportunity to reflect on, test, or demonstrate their knowledge and views [63]. Interactive activities, including assessments of learning and personal reflection activities, offer users the opportunity to reflect and reinforce their learning and become active participants in their education rather than passive consumers of information. Multimedia Appendices 1 and 2 display the engaging interactive activities that were acceptable to eTRIO users, which may be used in other web-based learning interventions and resources.

For both the clinician and patient-carer modules, we also incorporated a variety of media (text, audio, video, graphics, and images to cater to different learning styles and preferences. There is evidence suggesting that the use of multimedia may increase user satisfaction, acceptability, and engagement [64,65] and thus may improve adherence and broad implementation. The modules were designed such that users could navigate through them at their own pace and read, view, and explore the sections in a self-directed manner based on how they like to engage with and process content. For example, we found that users had mixed responses to the videos embedded in the training module. Some users commented that the videos were very long and that they would mentally switch off or skip them. Others claimed to be “visual learners” and thoroughly enjoyed the opportunity to observe scenarios in this format, especially because the videos included interactive “trigger” questions such as “What would you do next?*”* where they were required to apply some of their learning to a scenario*.* This approach has been used in other web-based health interventions [66,67], which include complementary text, images, videos, audios, and interactive content to convey the educational content and cater to these diverse user preferences.

### Strengths and Limitations

A thoughtful process of iterative design was conducted over a 2-year period, ultimately producing a suite of web-based interventions intended to improve communication between cancer clinicians, patients, and carers. However, important limitations should be noted. While extensive end-user feedback was collected through iterative feedback from clinician and consumer advisory groups, the sample size of participants (patients with cancer and carers) naive to the modules in phase 3 was small, and there was limited diversity among consumer advisers and participants. In addition, we did not measure the computer literacy of the participants, which may have impacted their views about the program’s usability. Thus, the attitudes and preferences of participants may not be reflective of the wider population. For example, we were unable to recruit a carer with low health literacy, and there was an overrepresentation of women.

Further usability and acceptability testing is currently underway in a larger study with a more diverse sample of patients and carers. Recruitment of participants in phase 3 was conducted through professional networks and social media, and therefore, the participants may have had a strong interest web-based learning or carer communication, which could have biased their views. This study focused only on development and user testing, and therefore, no assessment of the effectiveness or uptake of the modules has been conducted. Larger evaluation studies of the modules are currently being conducted, which will provide insight into the utility of the eTRIO modules in improving carer-related communication and inclusion.

Finally, while most patients with cancer have a carer or support person, some patients do not. Further studies are required to better understand the needs of people without a carer, which is beyond the scope of this study.

### Future Directions

The eTRIO and eTRIO-pc modules are now undergoing pre-post evaluation with additional qualitative learner feedback to inform the broad implementation and uptake of these educational resources.

### Conclusions

By including and being receptive to the needs of our user groups throughout the design process, we were able to create interventions that end users are likely to be more engaged and satisfied with.

## Appendix

Multimedia Appendix 1 e-Triadic Oncology patient and carer module features.

Multimedia Appendix 2 e-Triadic Oncology clinician module features.

## Abbreviations

CHESS: Comprehensive Health Enhancement Support System
eTRIO: e-Triadic Oncology
eTRIO-pc: e-Triadic Oncology for patients and carers
STREAM: Stress-Aktiv-Mindern
SUS: System Usability Scale
TRIO: Triadic Oncology
