# Supplementary material for: Development of Web-Based Education Modules to Improve Carer Engagement in Cancer Care: Design and User Experience Evaluation of the e-Triadic Oncology (eTRIO) Modules for Clinicians, Patients, and Carers
Source: JMIR Med Educ. 2024 Apr 17;10:e50118. doi: 10.2196/50118 (PMC11063882; doi:10.2196/50118)
Supplement: Multimedia Appendix 1 [file mededu_v10i1e50118_app1.docx]

Multimedia Appendix 1 – eTRIO Patient and Carer Module Features

| **Activities** | **Image** | **Description** |
| --- | --- | --- |
| Support team list building activity | 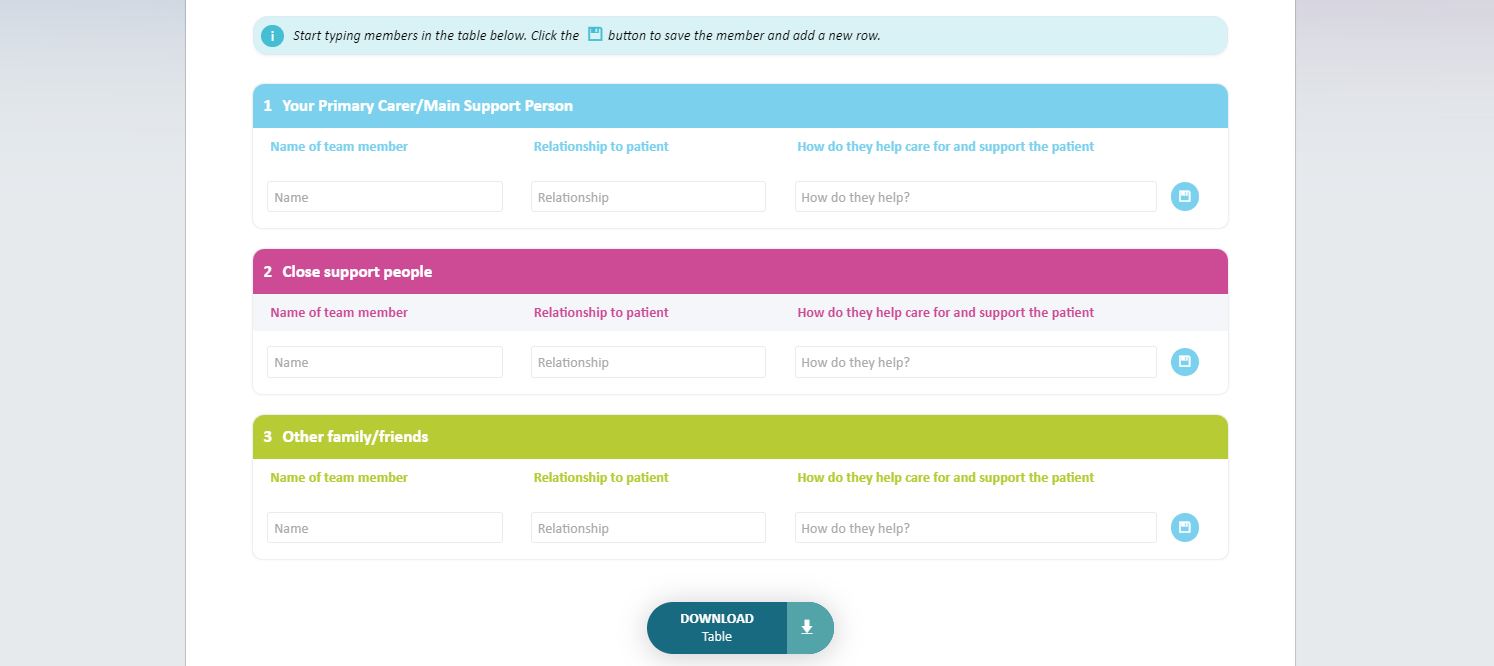 | Users can create a list of people in their support team, with option to download one’s personalised list |
| Question prompt list building activity | 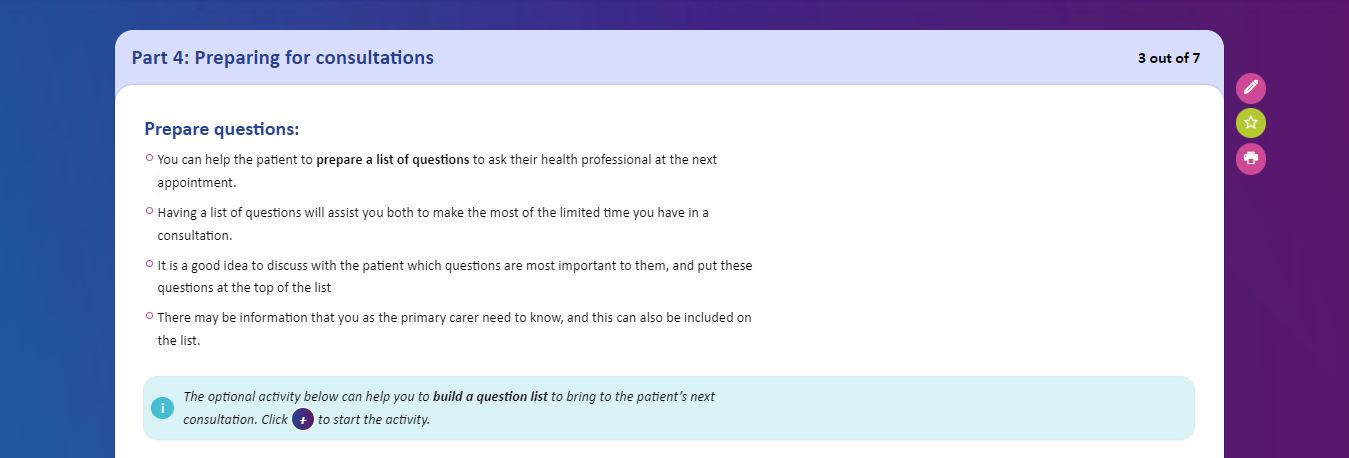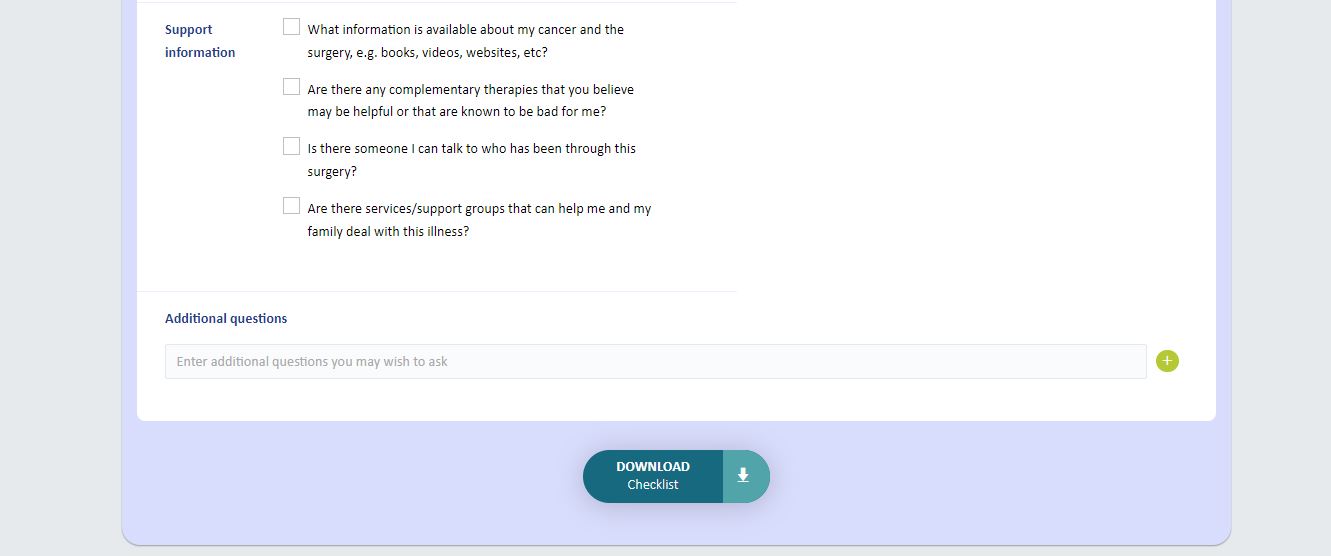 | Users can create a list of questions for different health professionals. Users check the boxes next to questions they would like to ask, can add additional questions not provided, and can download their final list. |
| Drag and Drop | 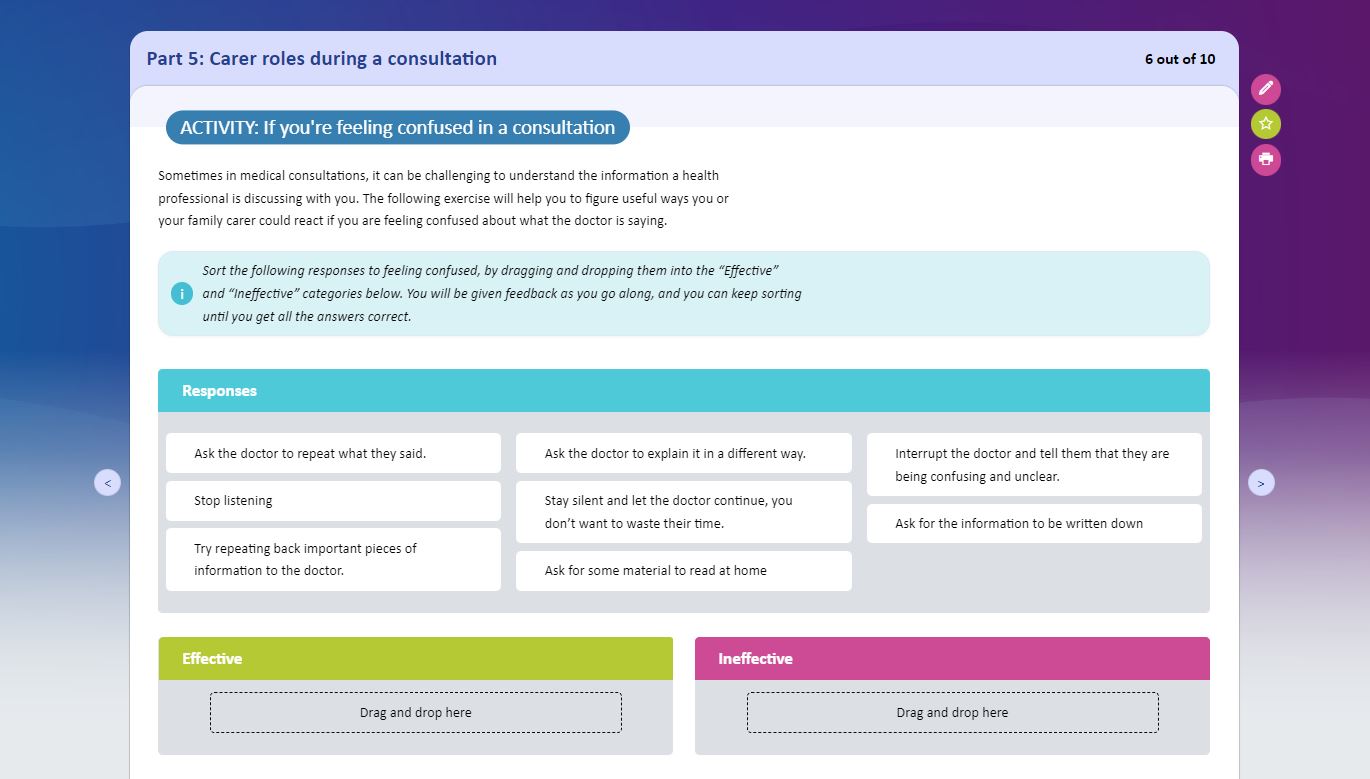 | Activity to help users learn communication strategies. Feedback is provided |
| Emotional check-in activity | 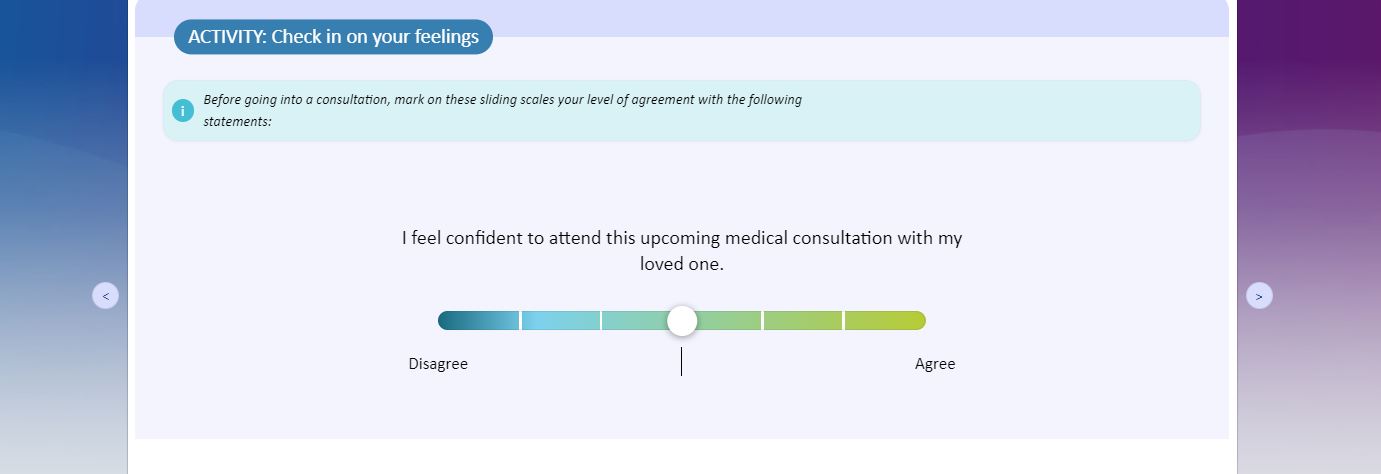 | Slider activity, users drag the slider as they reflect on their feelings |
| Reflection exercises/ open ended responses | 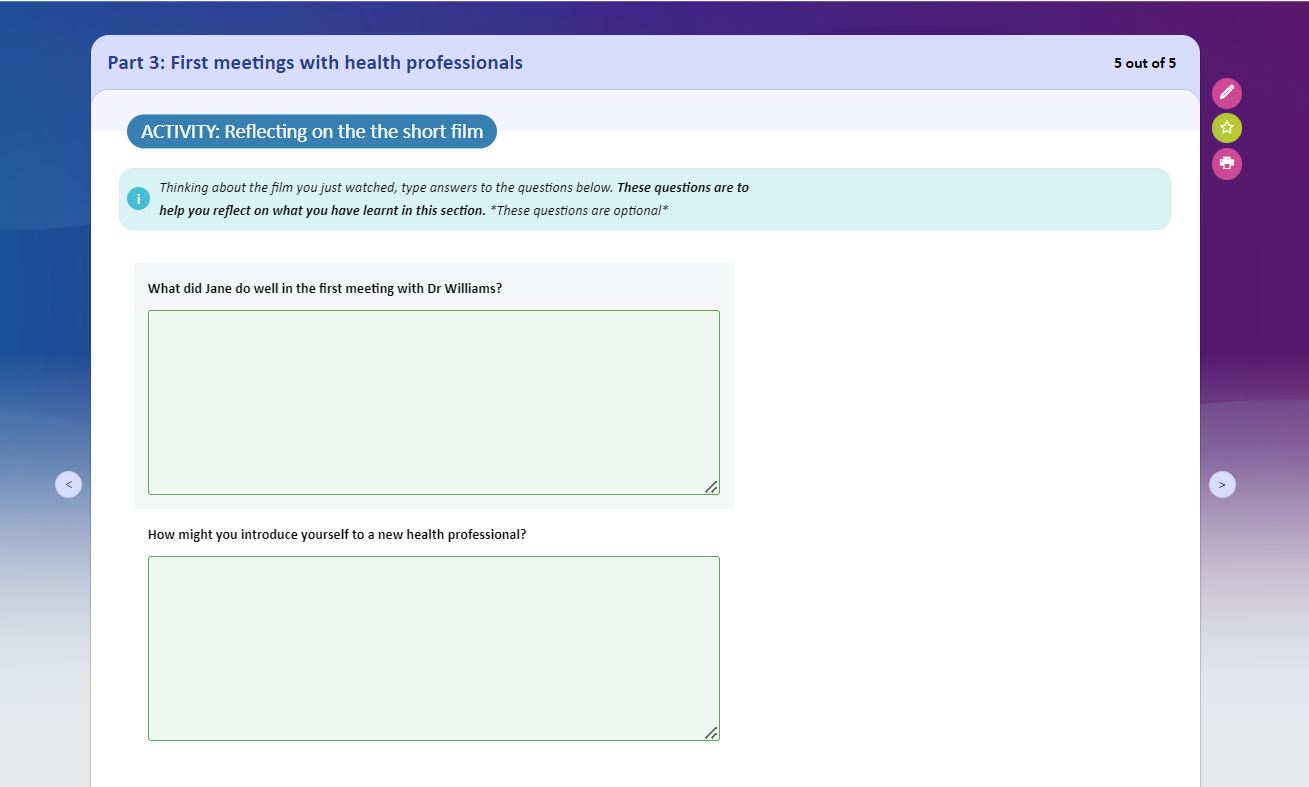 | Users are invited to write short responses to reflect and practice using the skills presented in the training |
| Click to reveal information | 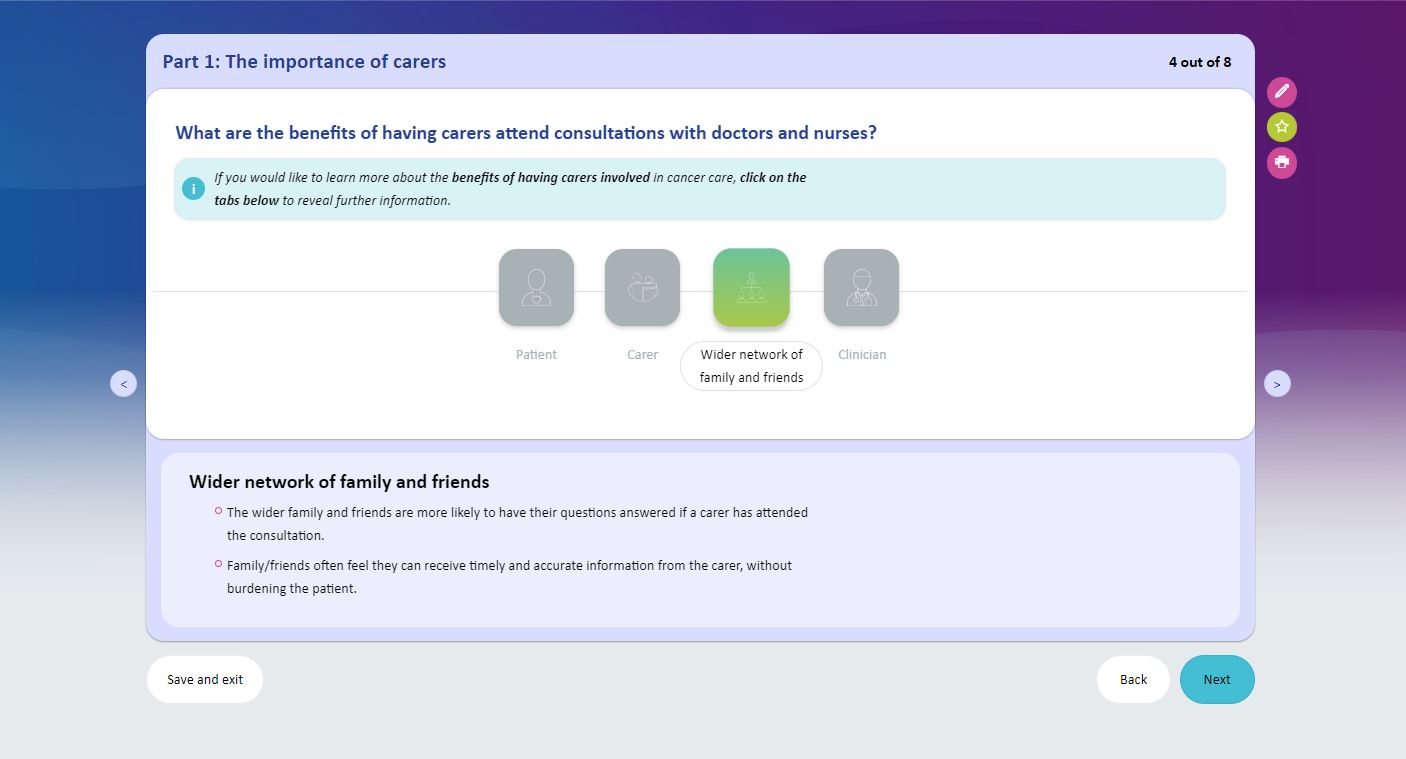 | Users can click on icons to reveal additional information. Users find this more engaging than reading plain text |
| “pop the bubbles” myth busting activity | 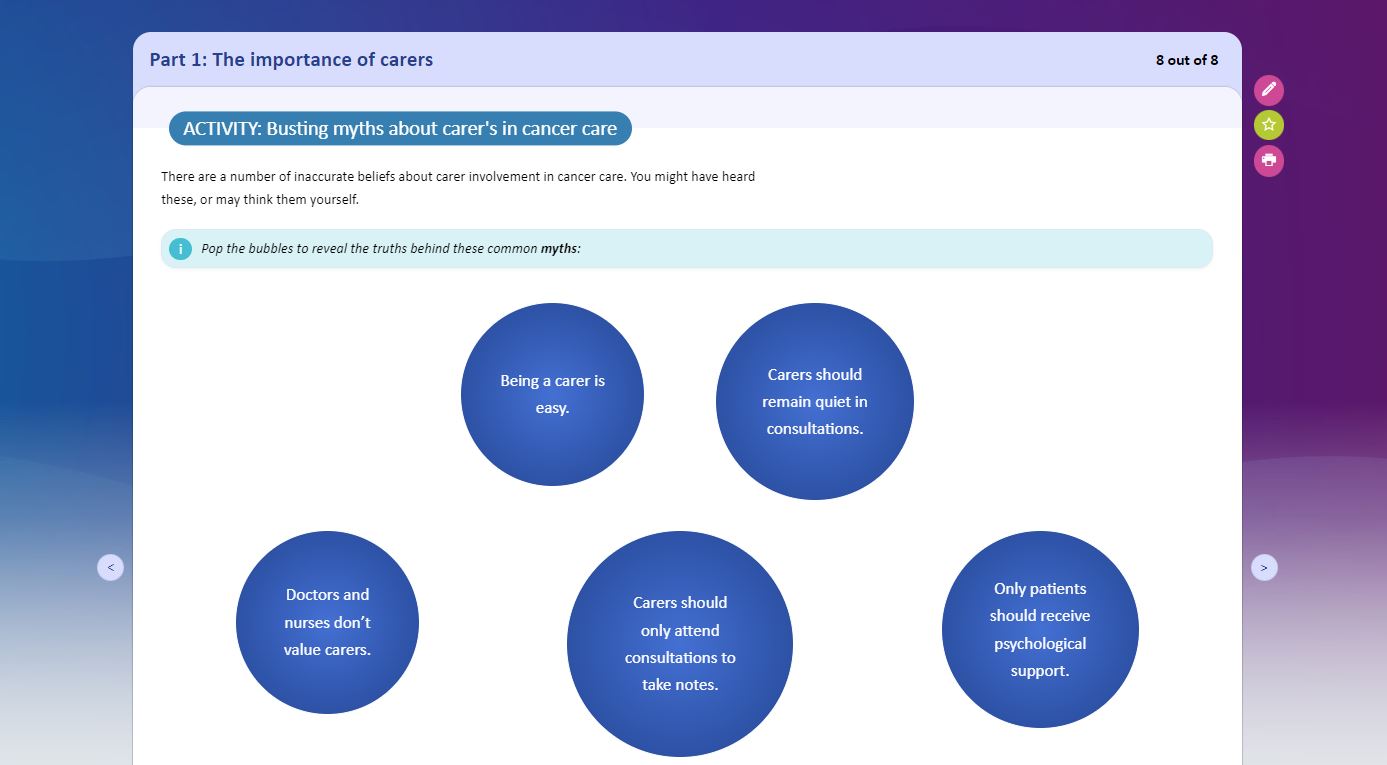 | An interactive activity where users can reflect on their attitudes. Information is presented in an engaging and interactive way |

| **Design Feature** | **Image** | **Description** |
| --- | --- | --- |
| Navigation instructions | 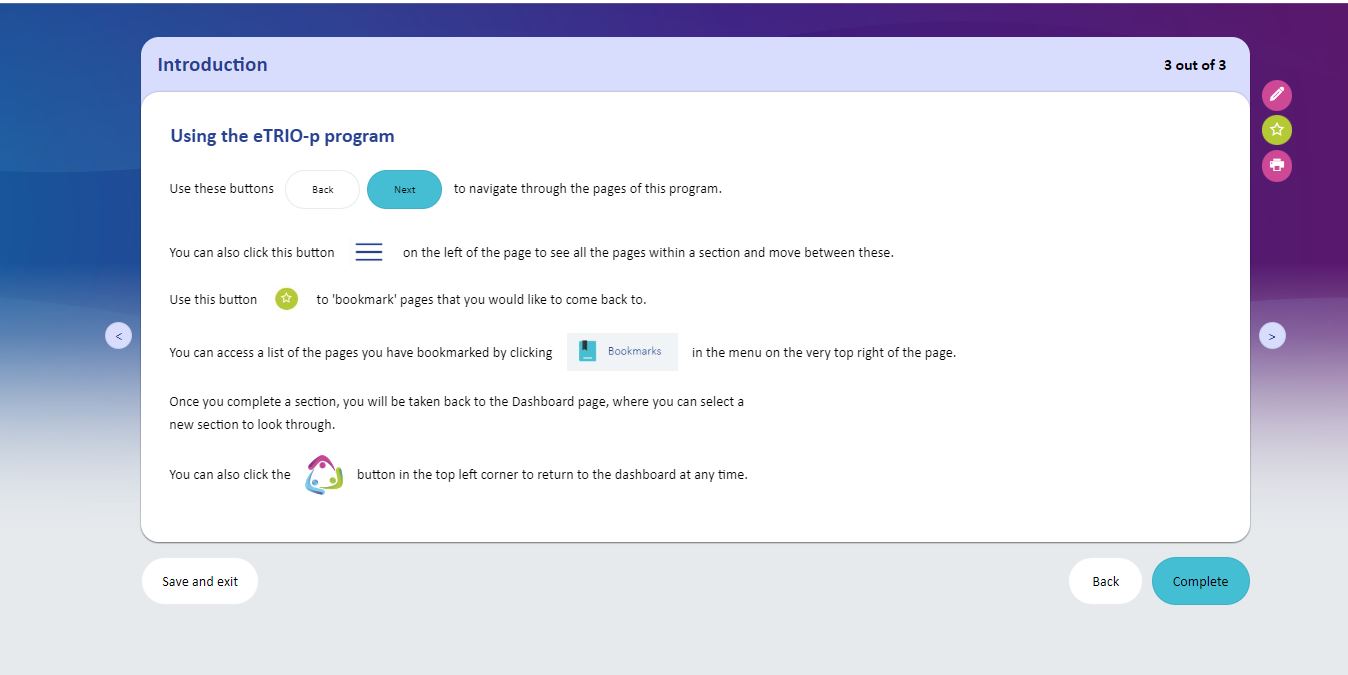 | Users are provided with instructions for how to navigate through the module |
| Section Aims | 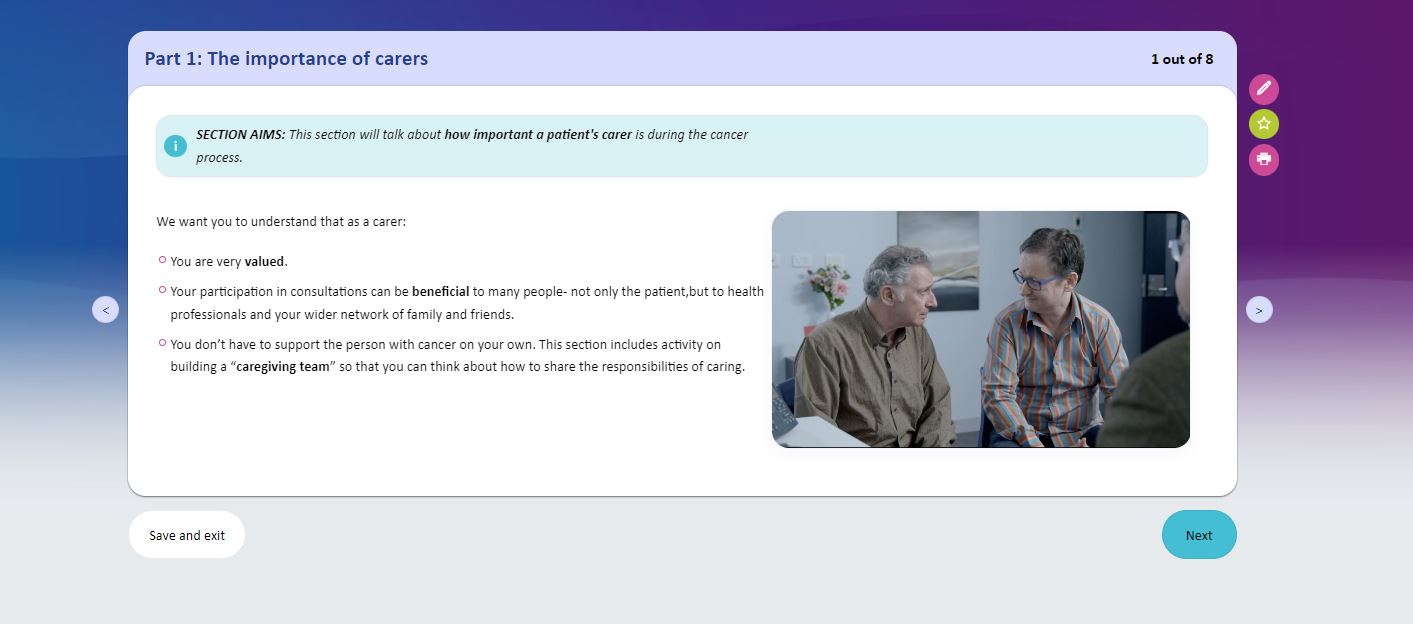 | Each section begins with a concise aim and learning objectives |
| Expandable content | 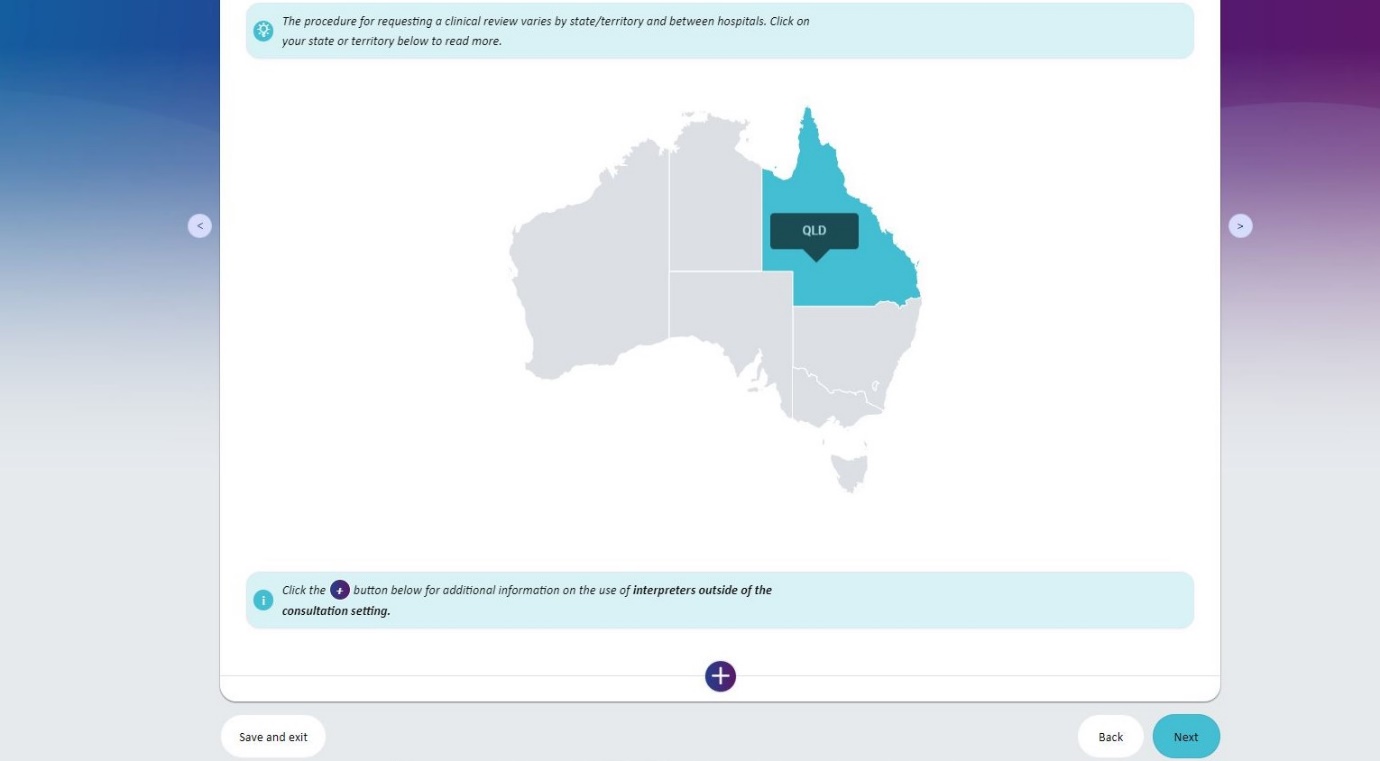 | For those wanting extra information. Navigational instructions appear in pale blue boxes |
| Navigation Buttons | 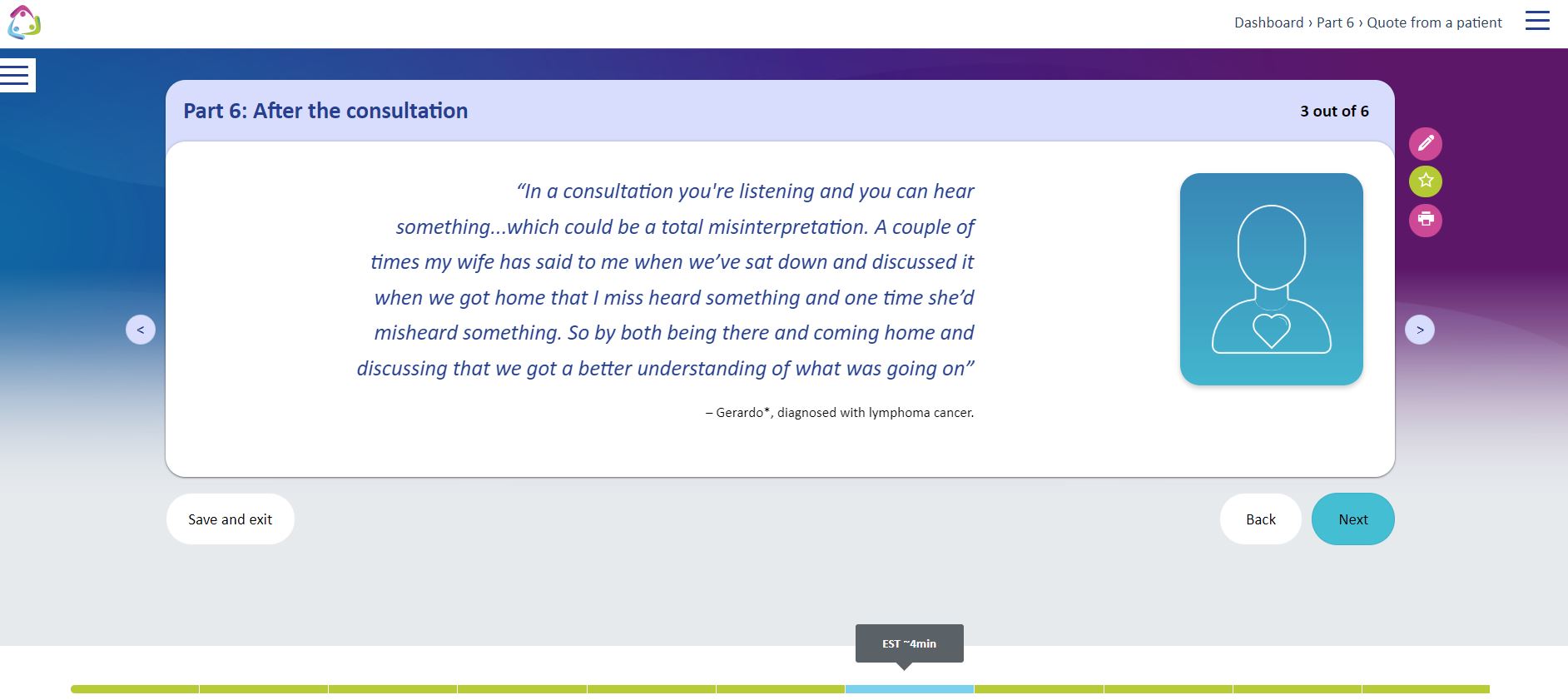 | “Back”, “Next”, “Save and exit” buttons, arrow buttons, progress bar with estimated time remaining to complete the section |
| Videos and vignettes | 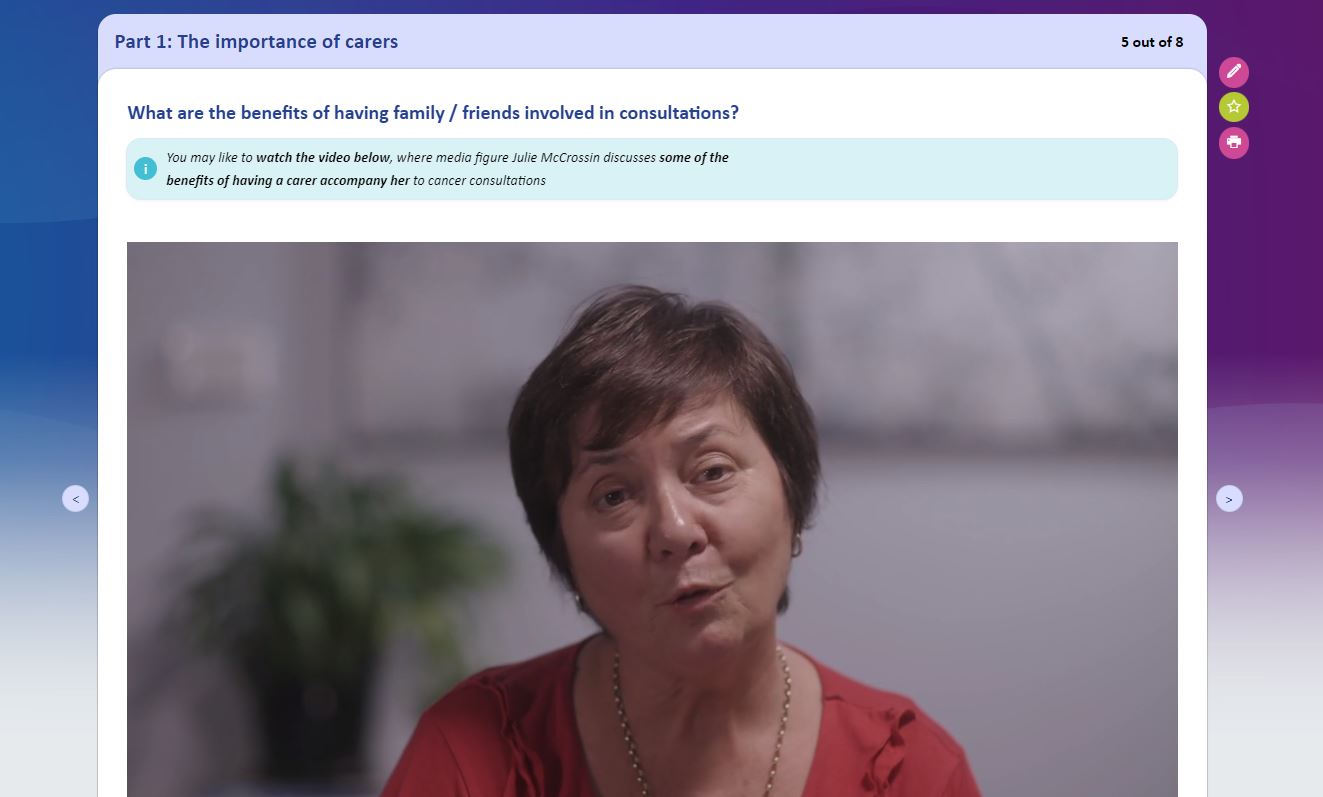 | Video are included to provide multi-modal content. Videos can easily be played, paused and rewound |
| Text formatting | 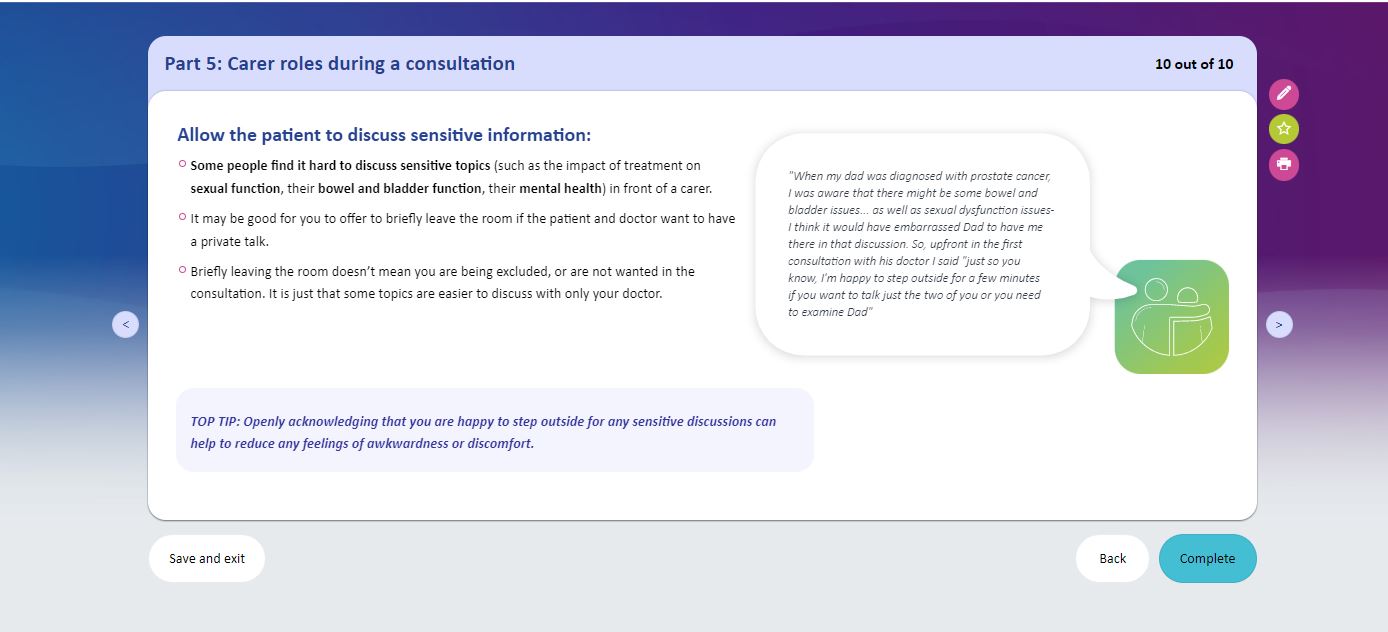 | Concise use of text. Coloured text, text boxes, salient points bolded, and use of bullet points |
| Quotes | 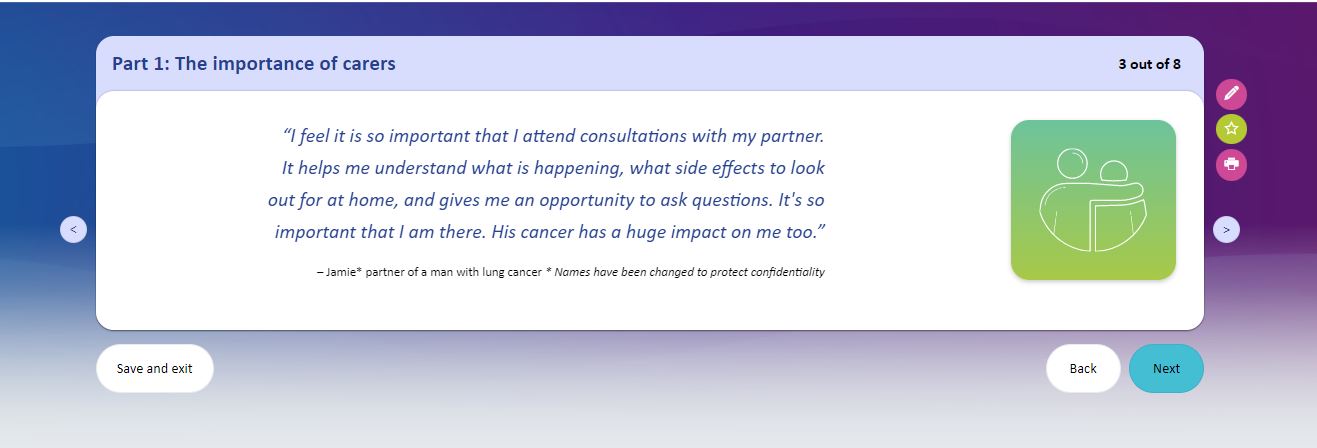 | Words from real patients and carers are provided to make content relatable |
| Illustrative images | 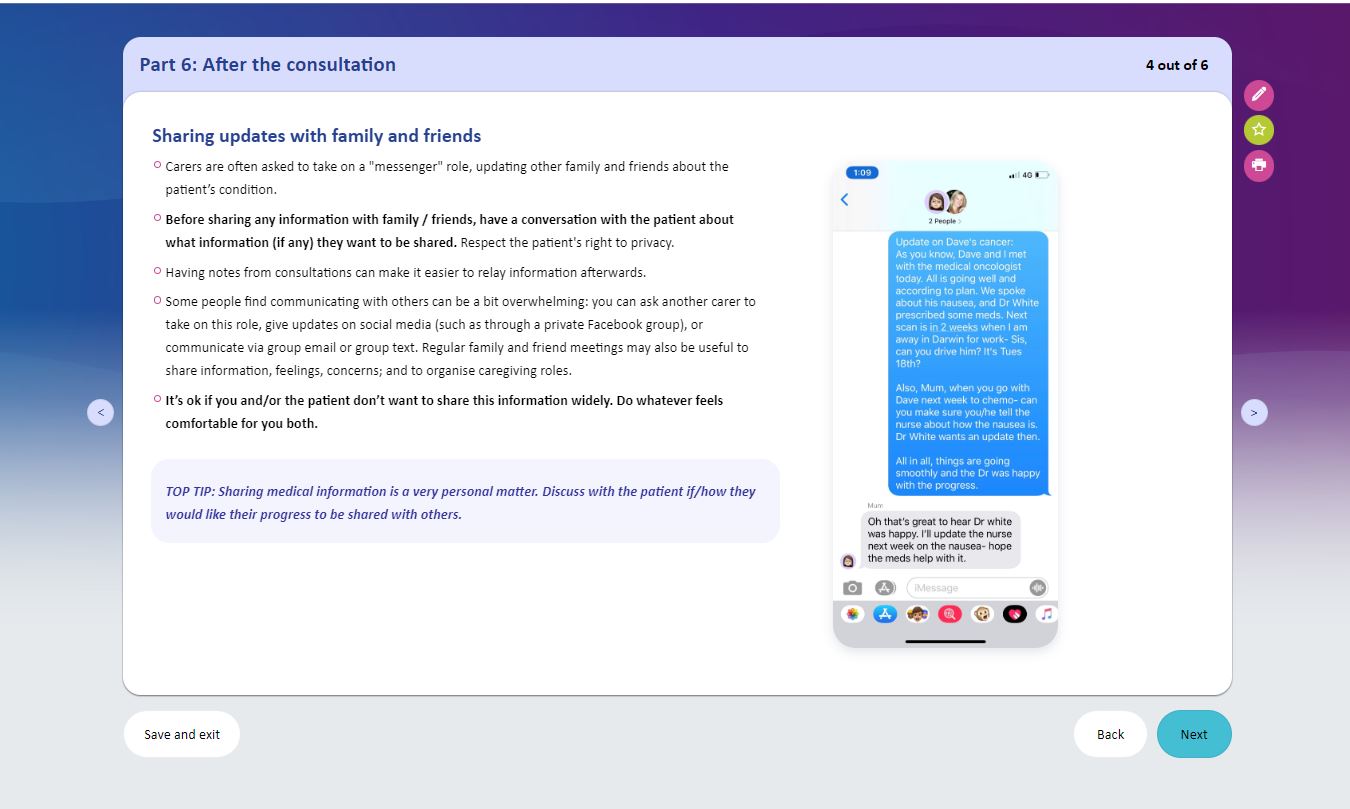 | Images used to demonstrate suggested strategies |
