# Supplementary material for: Development of Web-Based Education Modules to Improve Carer Engagement in Cancer Care: Design and User Experience Evaluation of the e-Triadic Oncology (eTRIO) Modules for Clinicians, Patients, and Carers
Source: JMIR Med Educ. 2024 Apr 17;10:e50118. doi: 10.2196/50118 (PMC11063882; doi:10.2196/50118)
Supplement: Multimedia Appendix 2 [file mededu_v10i1e50118_app2.docx]

Multimedia Appendix 2 – eTRIO Clinician Module Features

Table S1: Examples of eTRIO Interactive activities

| **Activity** | **Image** | **Description** |
| --- | --- | --- |
| Attitude reflection activity | 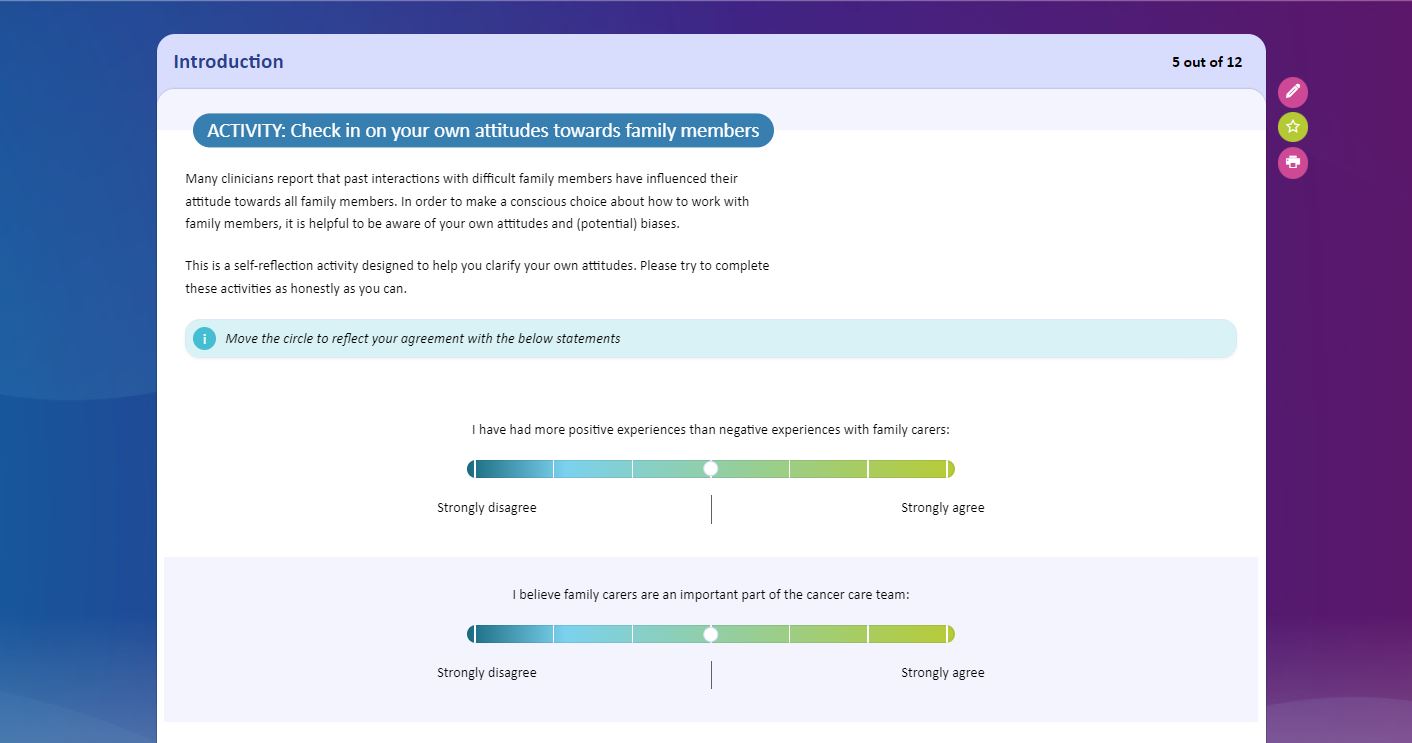 | Slider activity for users to reflect on their attitudes. |
| Click to reveal information | 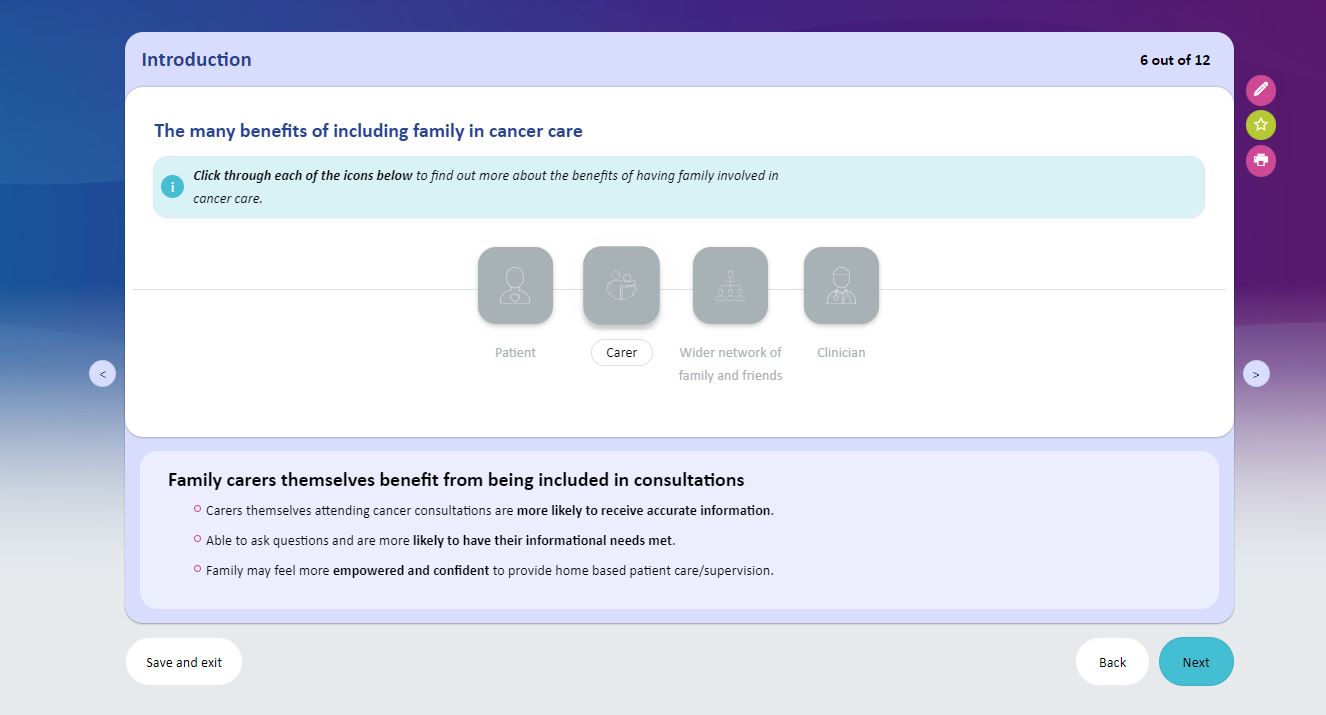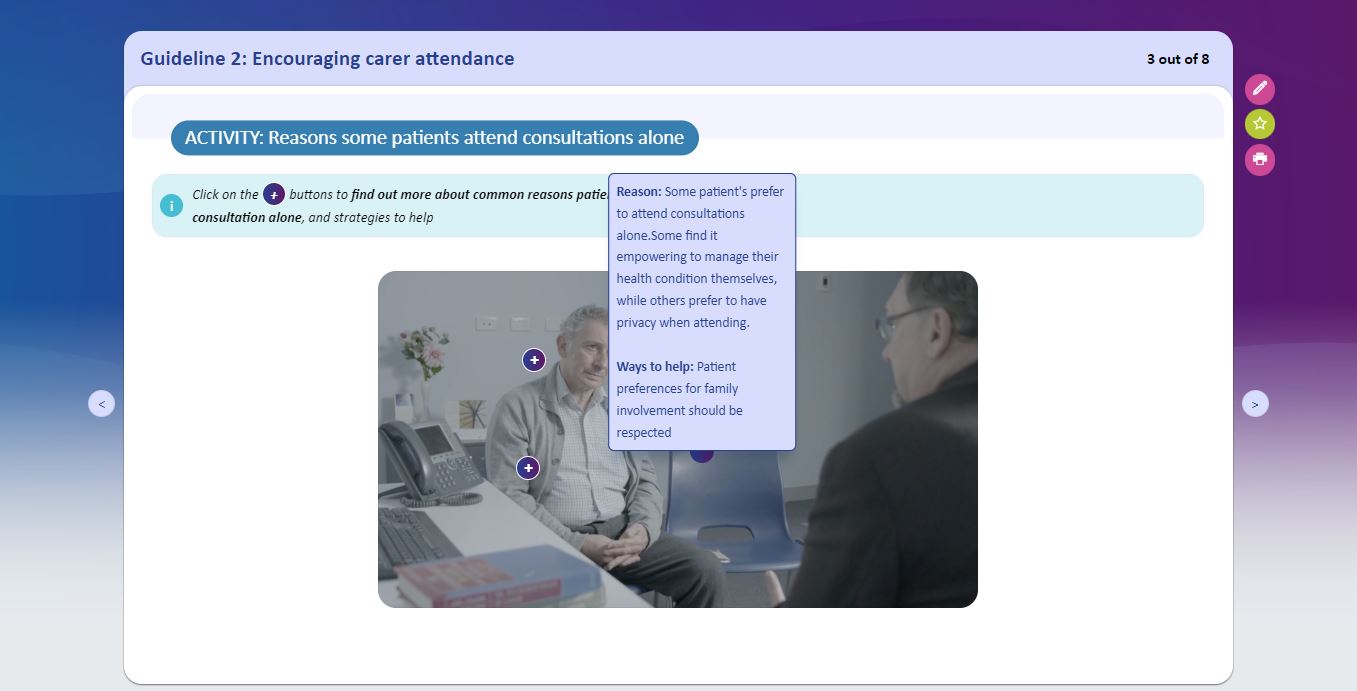 | Opportunities to click to reveal information provided a more interactive learning experience. |
| True or false activities | 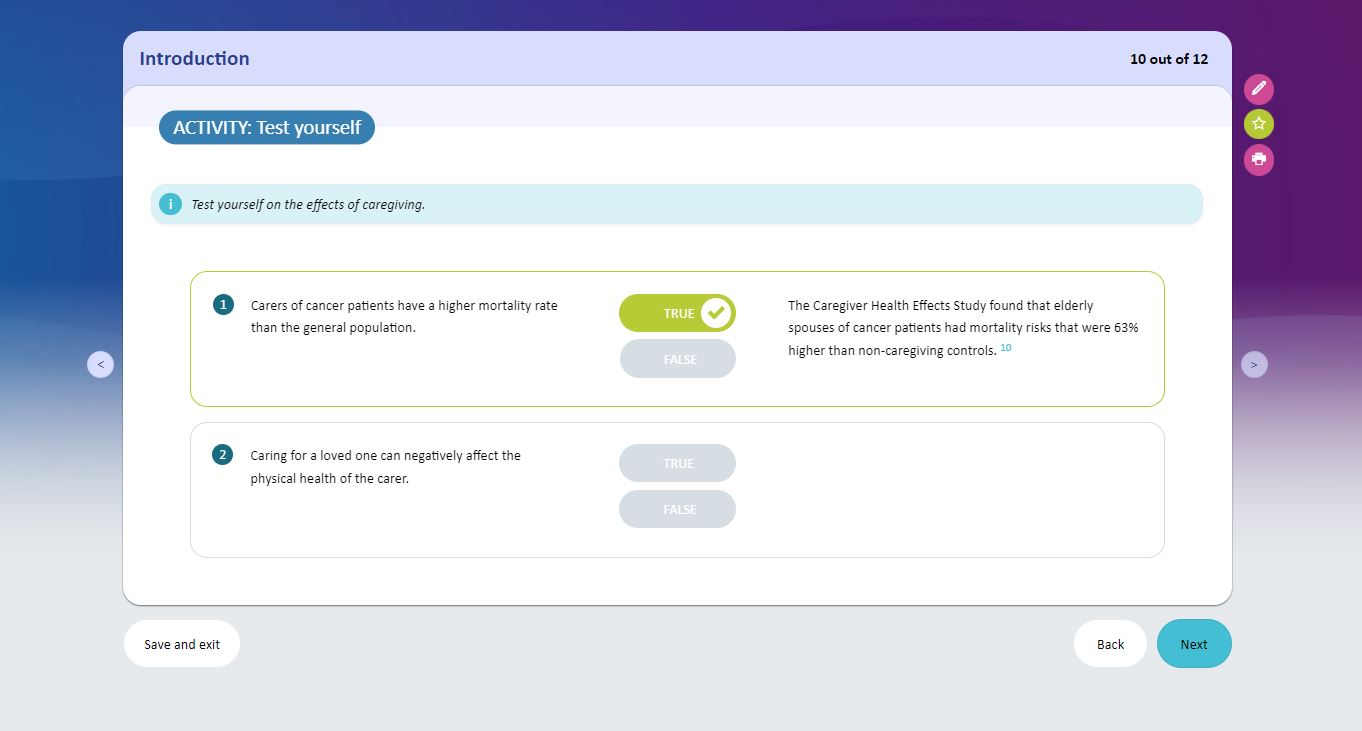 | True or false activities to test knowledge and understanding. Feedback is provided instantaneously. |
| Open-ended responses | 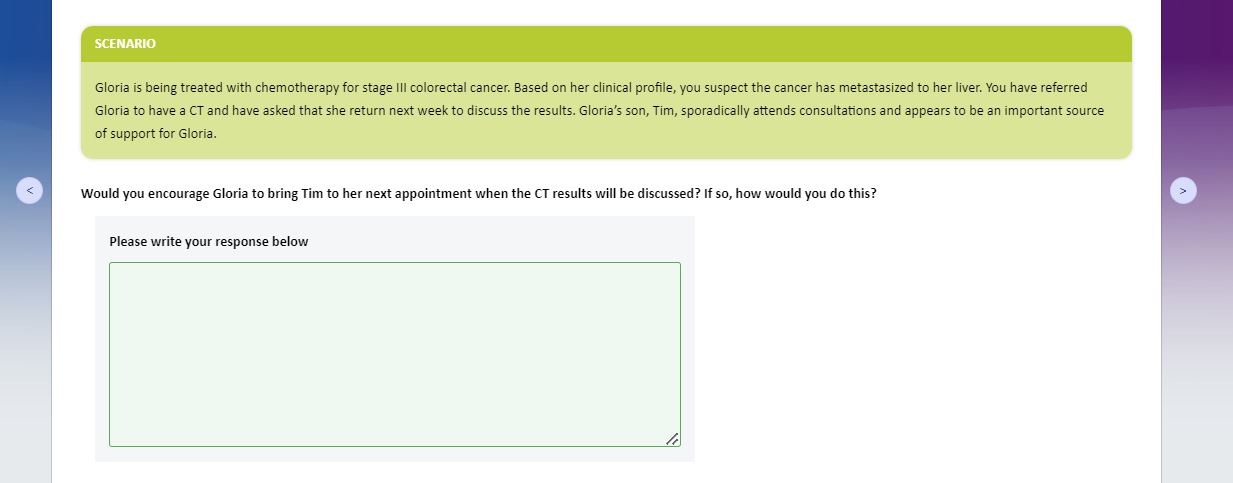 | Users are invited to reflect on how they would manage various scenarios. |
| Identifying communication strategies activity | 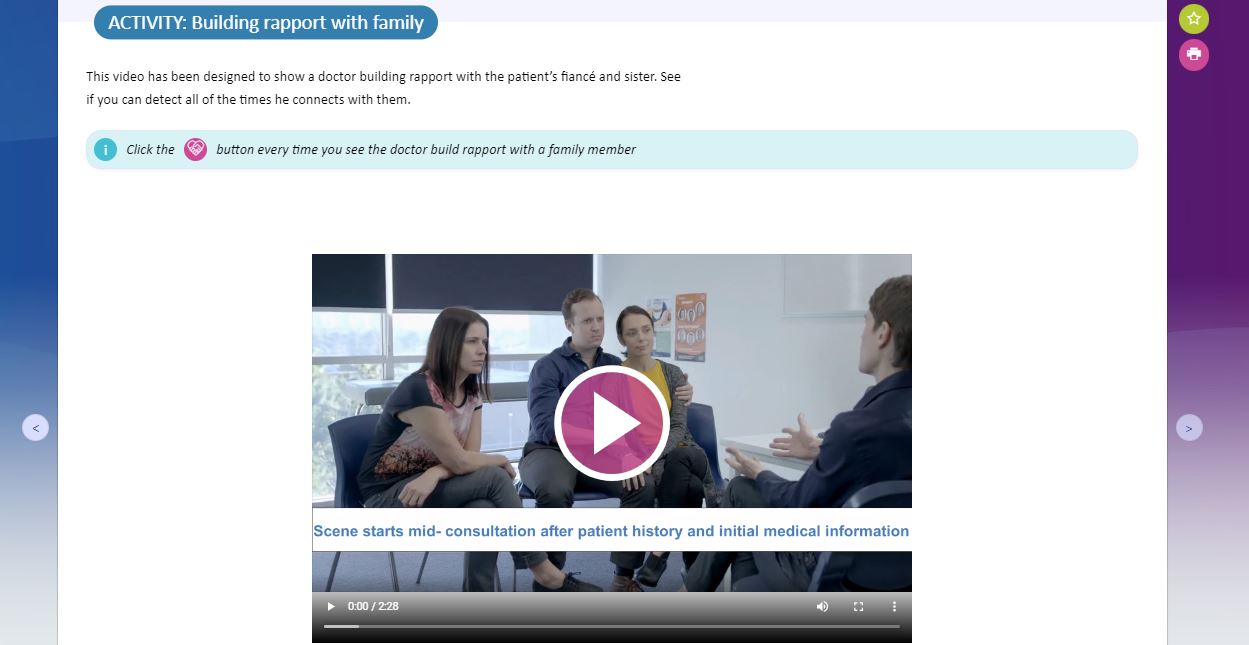 | Users are asked to identify communication strategies used in a video vignette by clicking when they observe these skills being used. Feedback is provided. |
| Multiple choice activities | 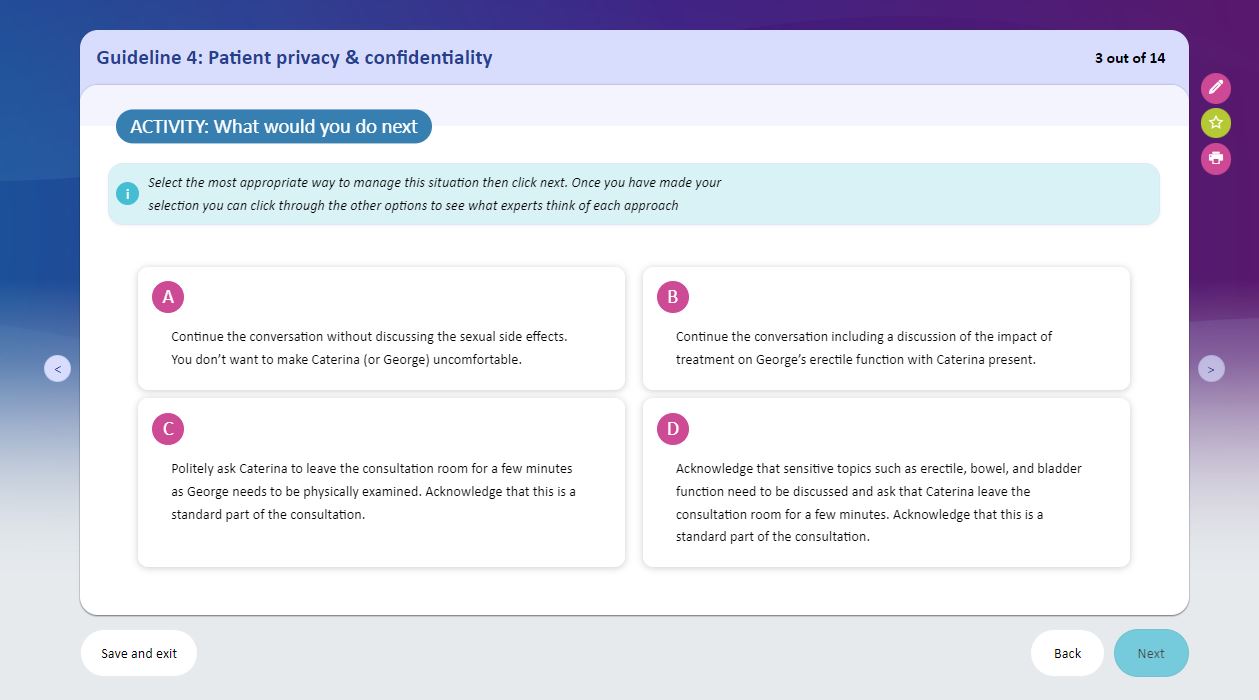 | Multiple choice activity to test learning. Feedback is provided. |

Table S2: Examples of eTRIO Design Features

| **Design Feature** | **Image** | **Description** |
| --- | --- | --- |
| Navigation instructions | 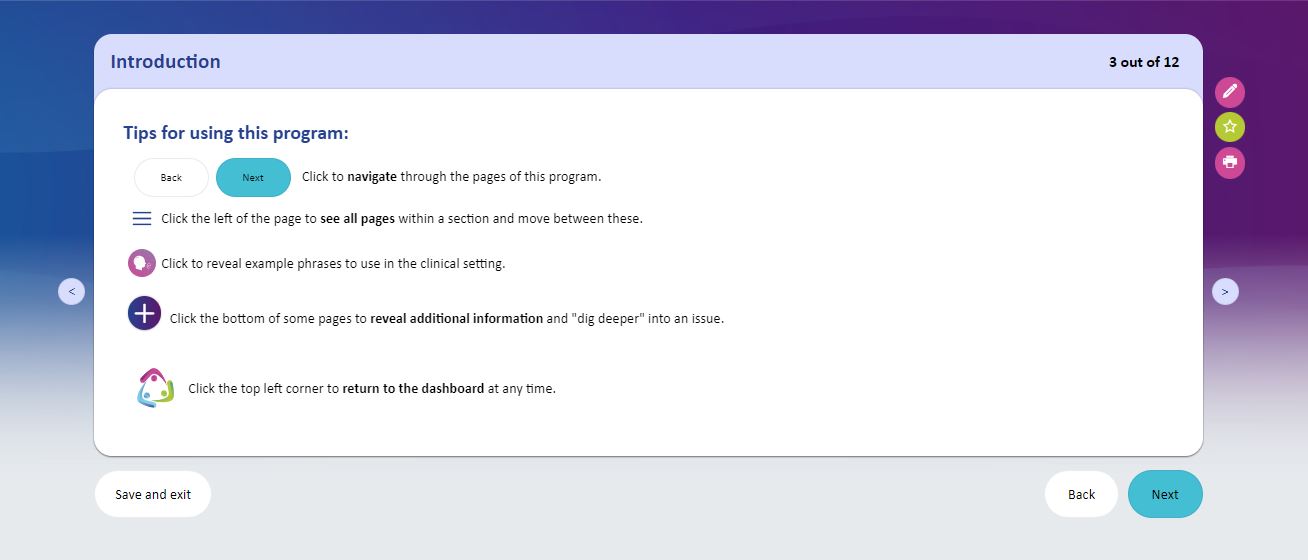 | Users are provided with instructions for how to navigate through the module |
| Learning outcomes are signposted | 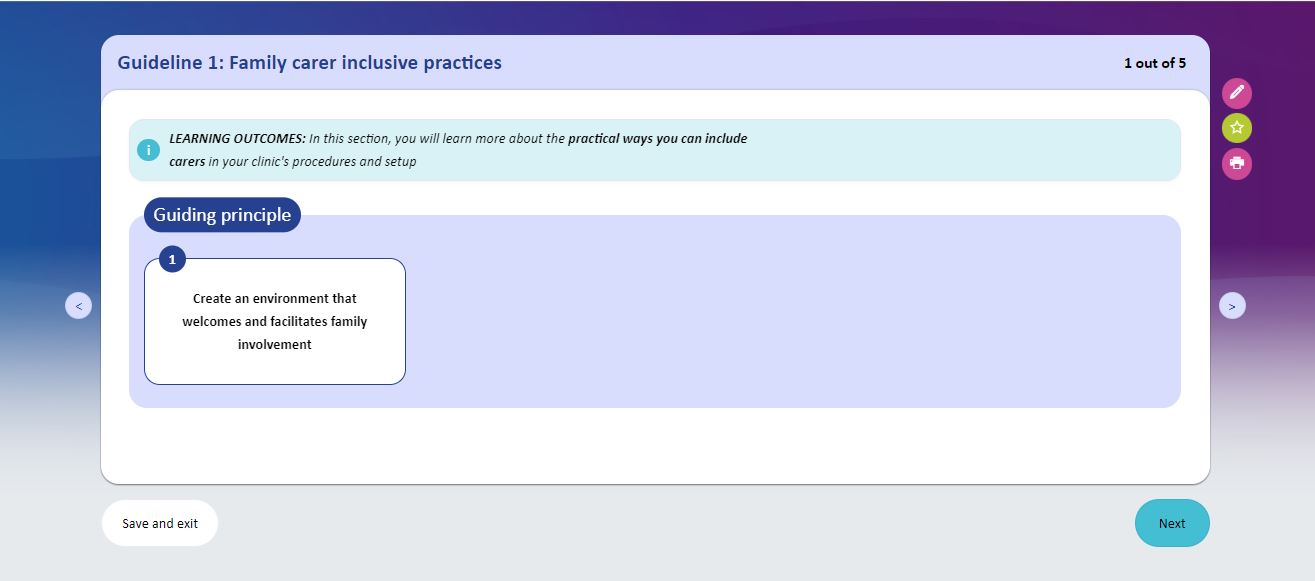 | Each section begins with a text signposting the learning objective and guiding principles that will be conveyed in the section. |
| Illustrative images | 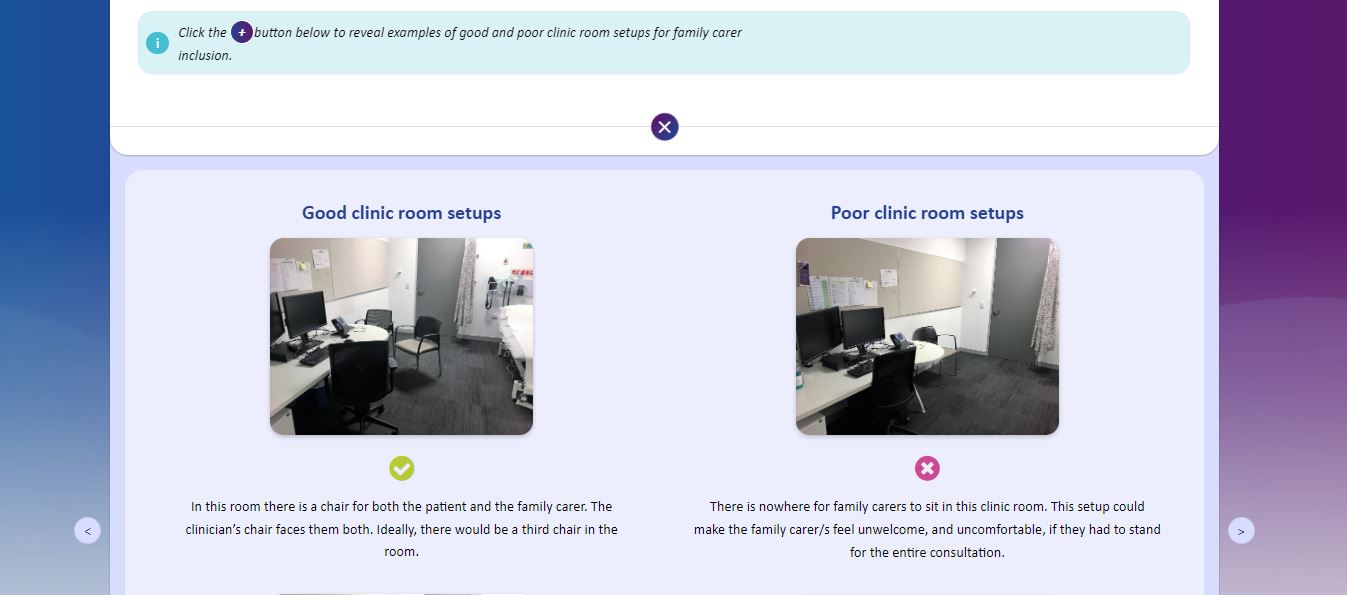 | Images used to demonstrate practical strategies to accommodate carers. |
| Video vignettes | 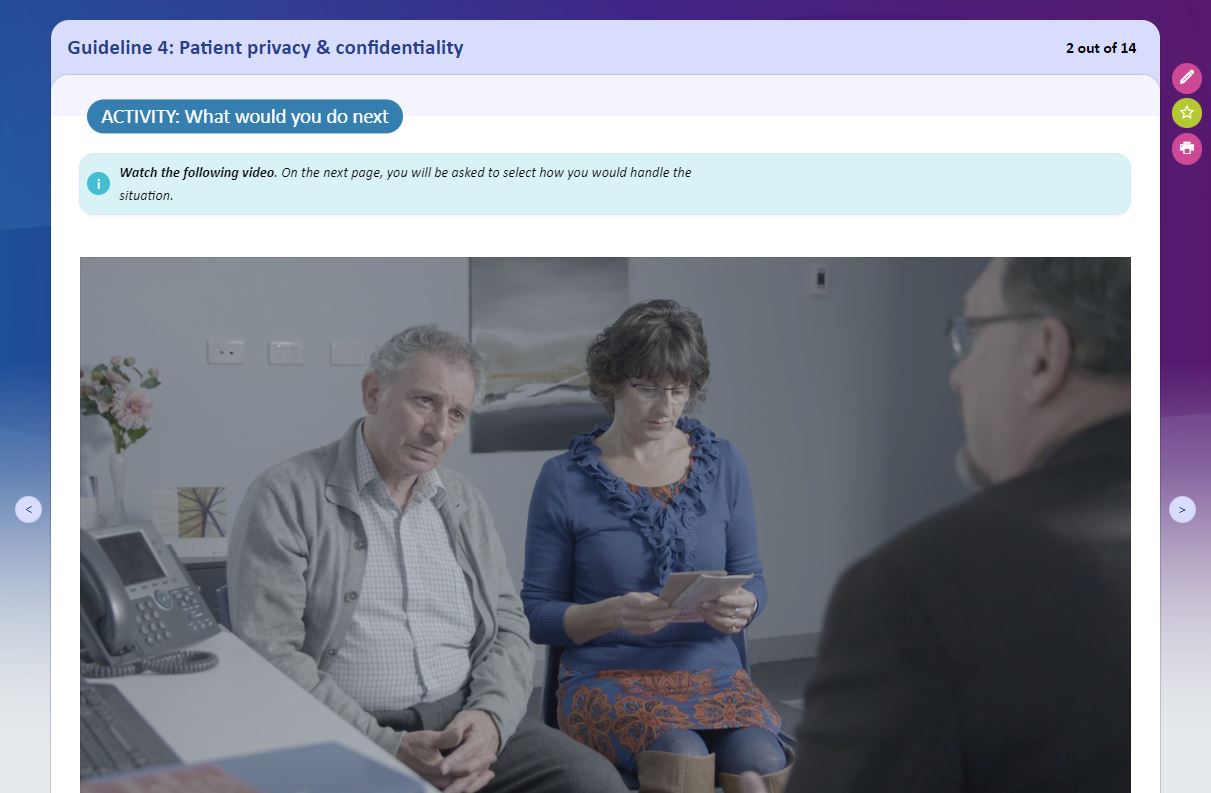 | Video are included to provide multi-modal content. Videos can easily be played, paused and rewound. |
| Text formatting | 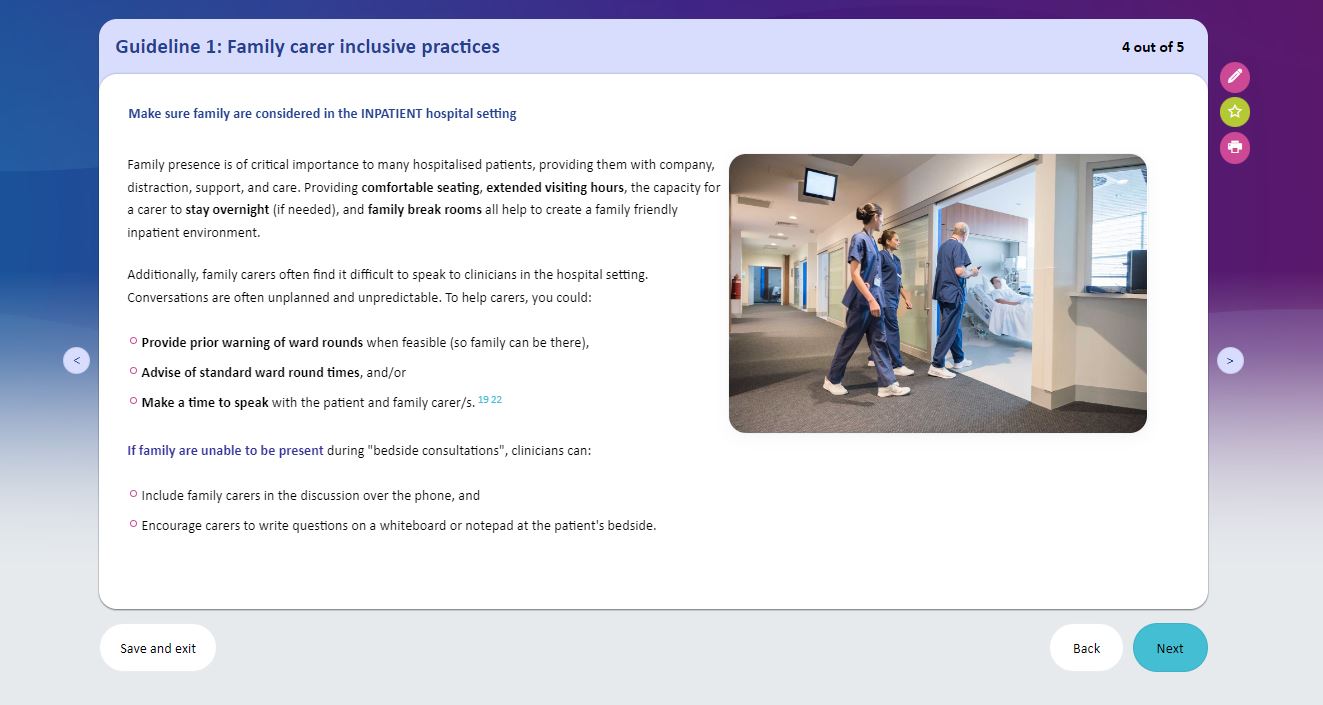 | Concise use of text. Coloured text, text boxes, salient points bolded, and use of bullet points. |
| Quotes | 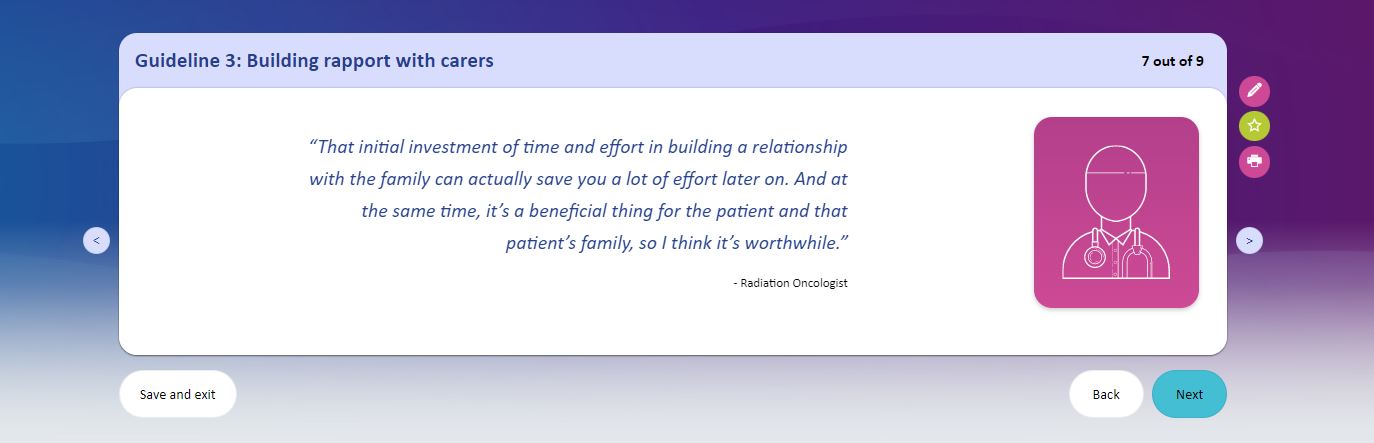 | Quotes from health professionals and academic journals are provided to reflect relevant ideas in a relatable format |
| Downloadable summaries | 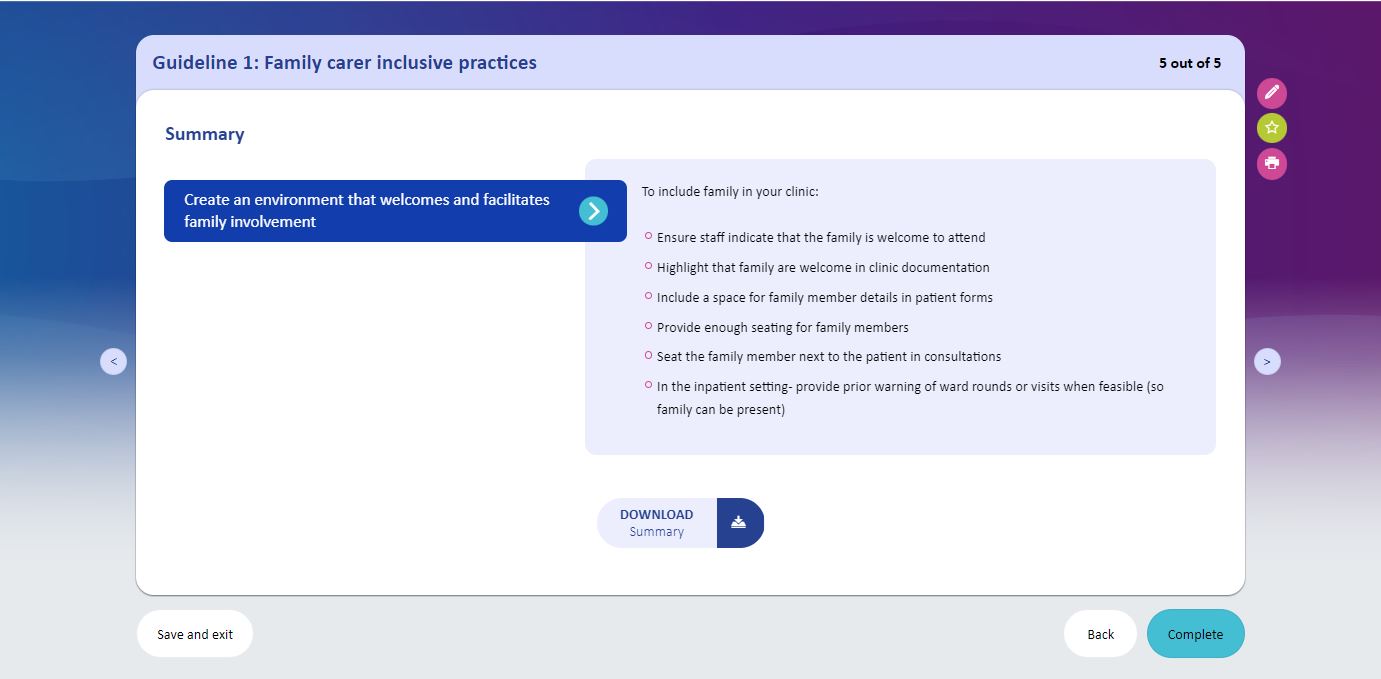 | At the end of each section summaries are provided and these can be downloaded by the user if desired. |
